# Supplementary material for: Small Molecules with Thiourea Skeleton Induce Ethylene Response in Arabidopsis
Source: Int J Mol Sci. 2023 Aug 4;24(15):12420. doi: 10.3390/ijms241512420 (PMC10418922; doi:10.3390/ijms241512420)
Supplement: Supplementary file 1 [file ijms-24-12420-s001.zip › ijms-2510761-supplementary.docx]

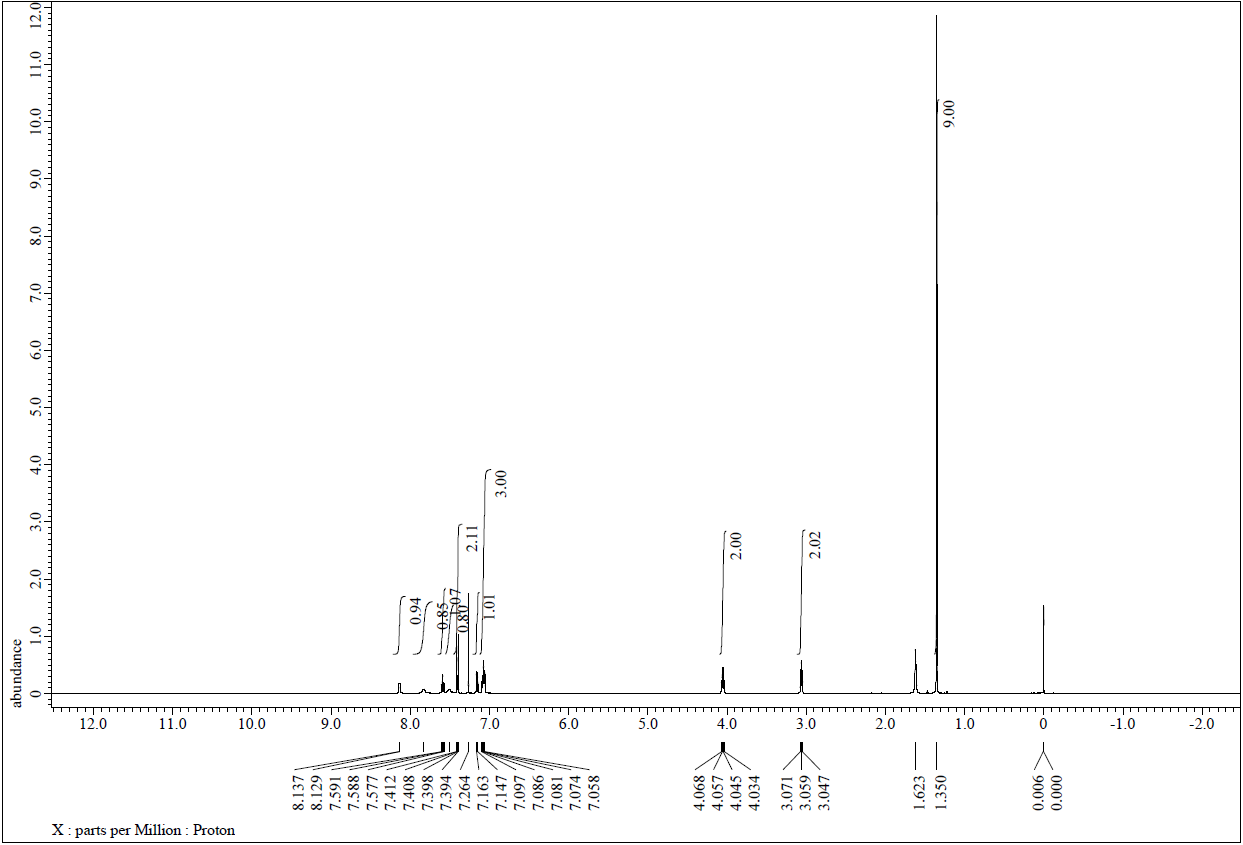


**Figure S1.** ^1^H spectrum for compound ZKT2.


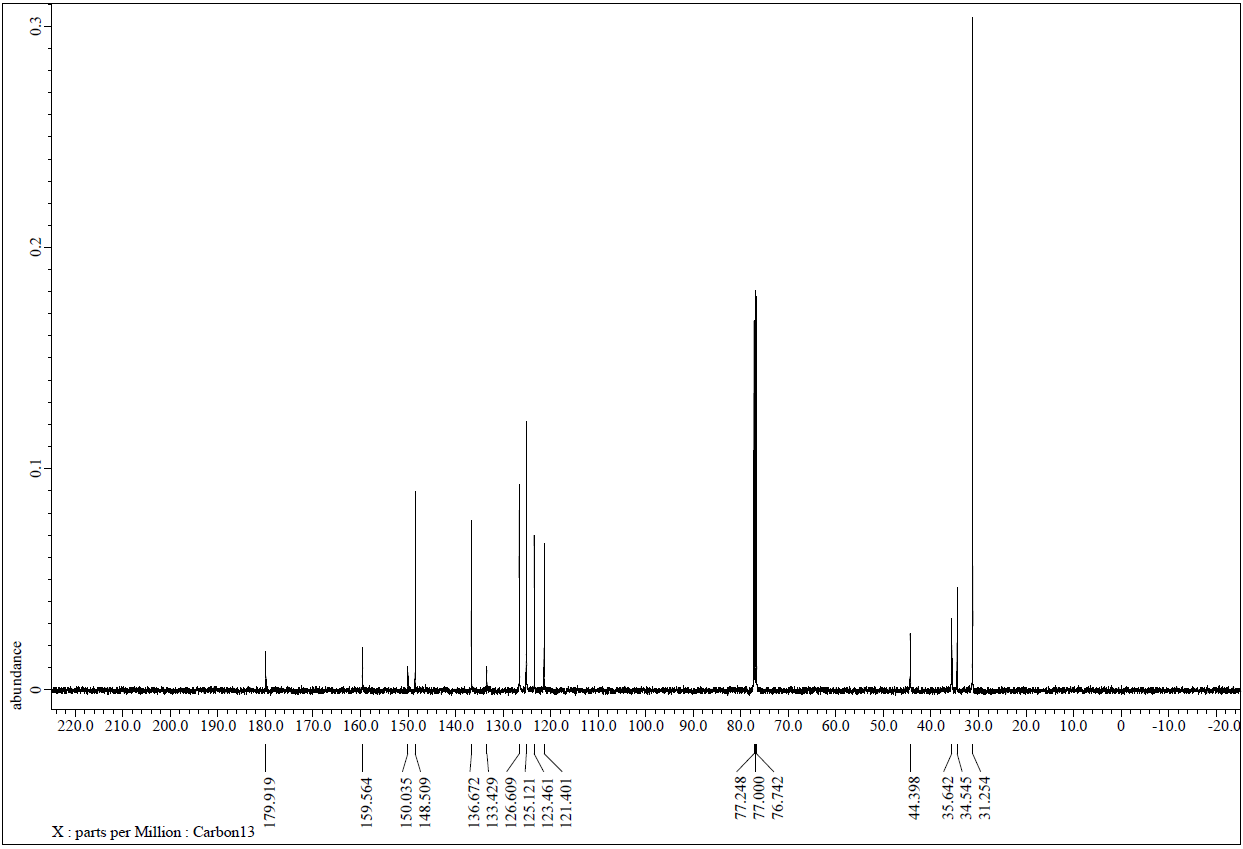


**Figure S2.** ^13^C NMR spectrum for compound ZKT2


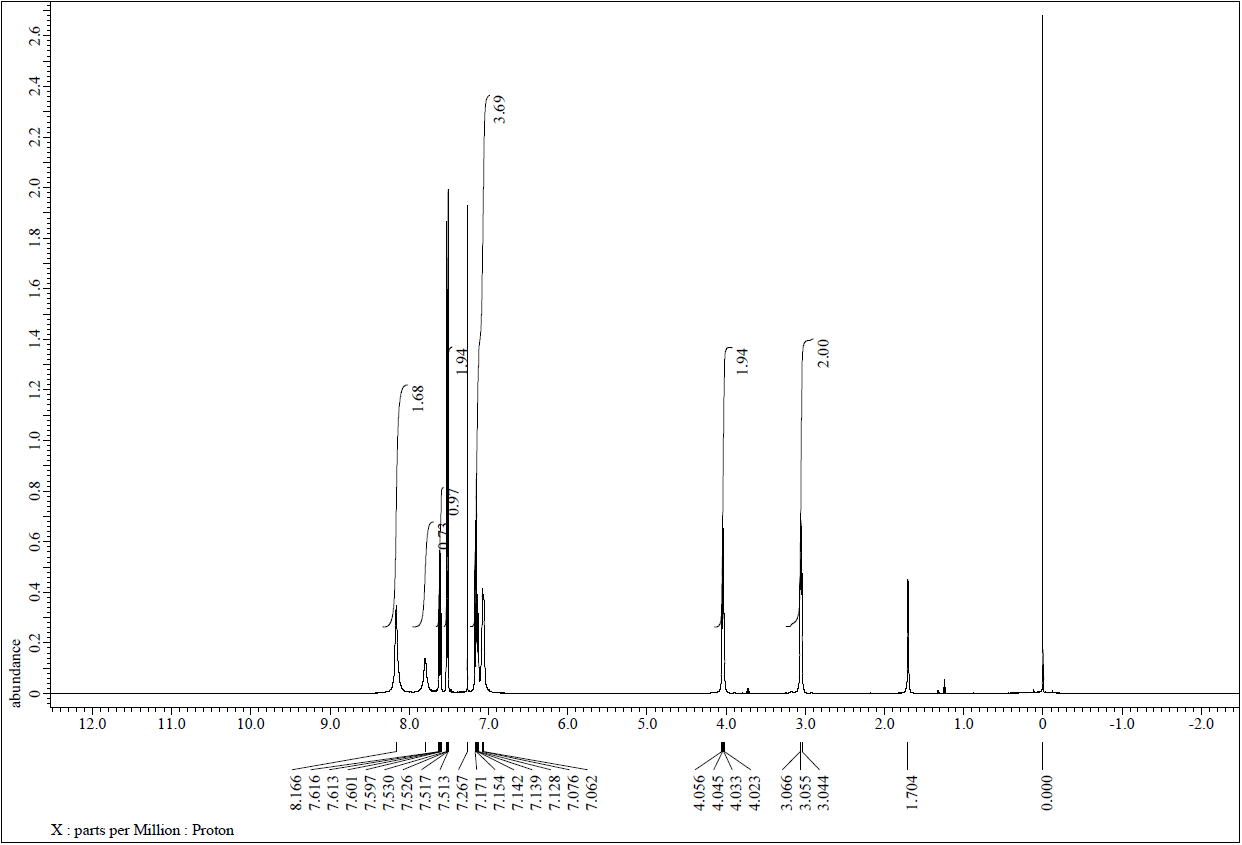


**Figure S3.** ^1^H spectrum for compound ZKT3.


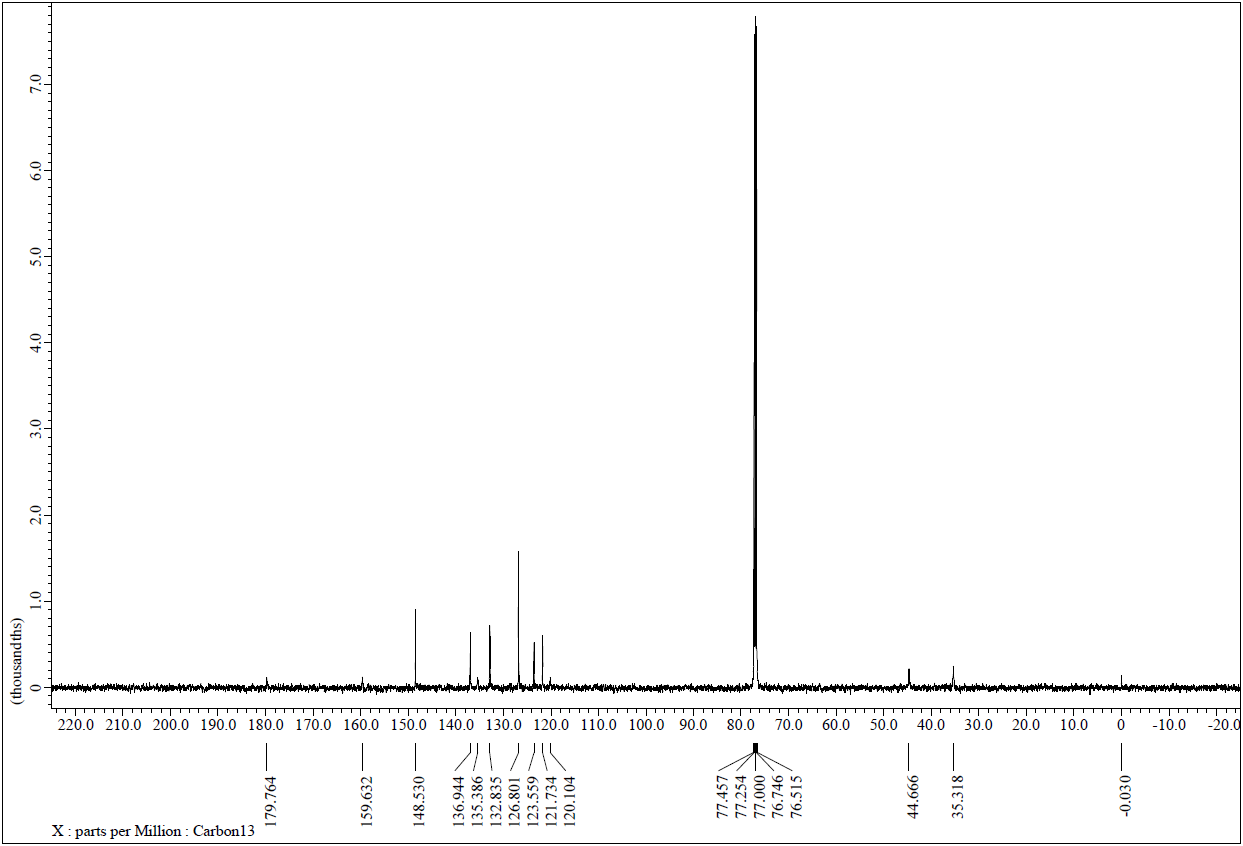


**Figure S4.** ^13^C NMR spectrum for compound ZKT3.


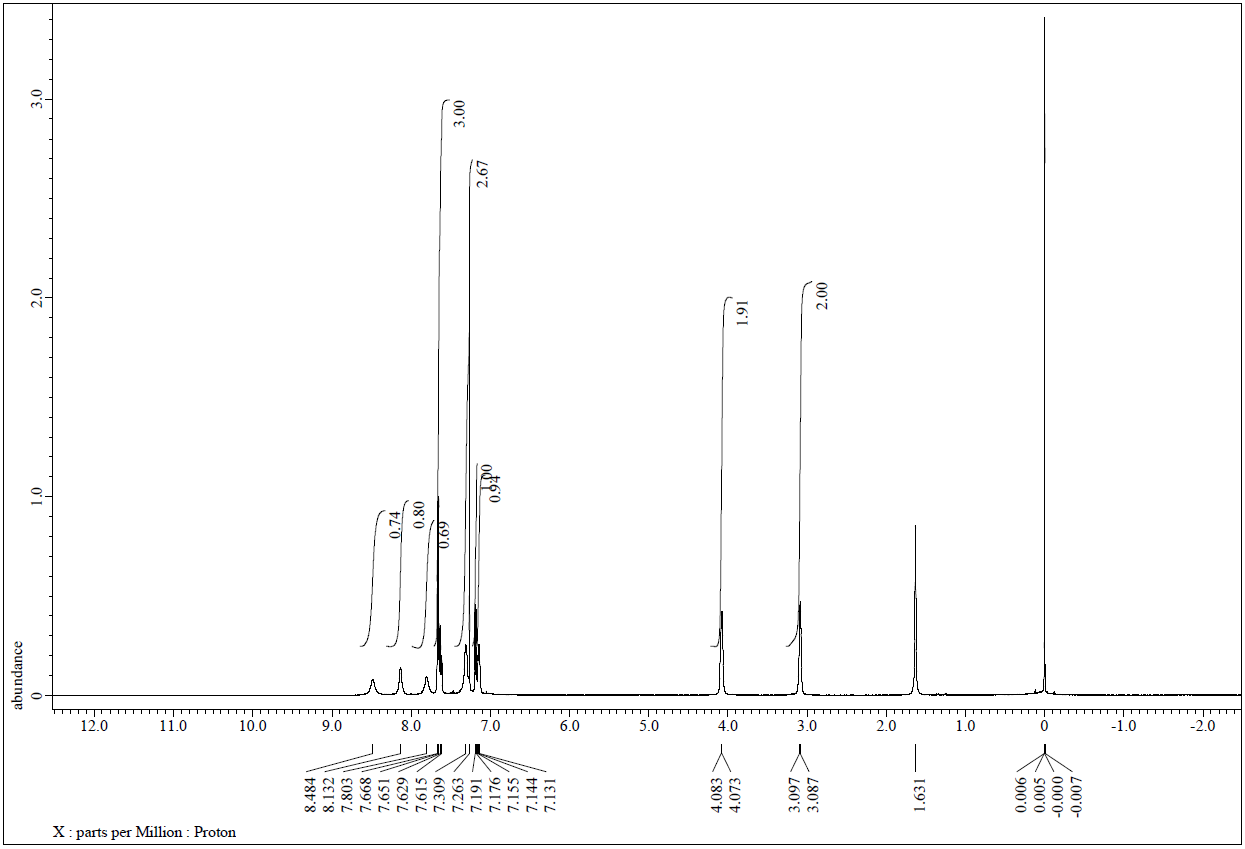


**Figure S5.** ^1^H NMR spectrum for compound ZKT4.


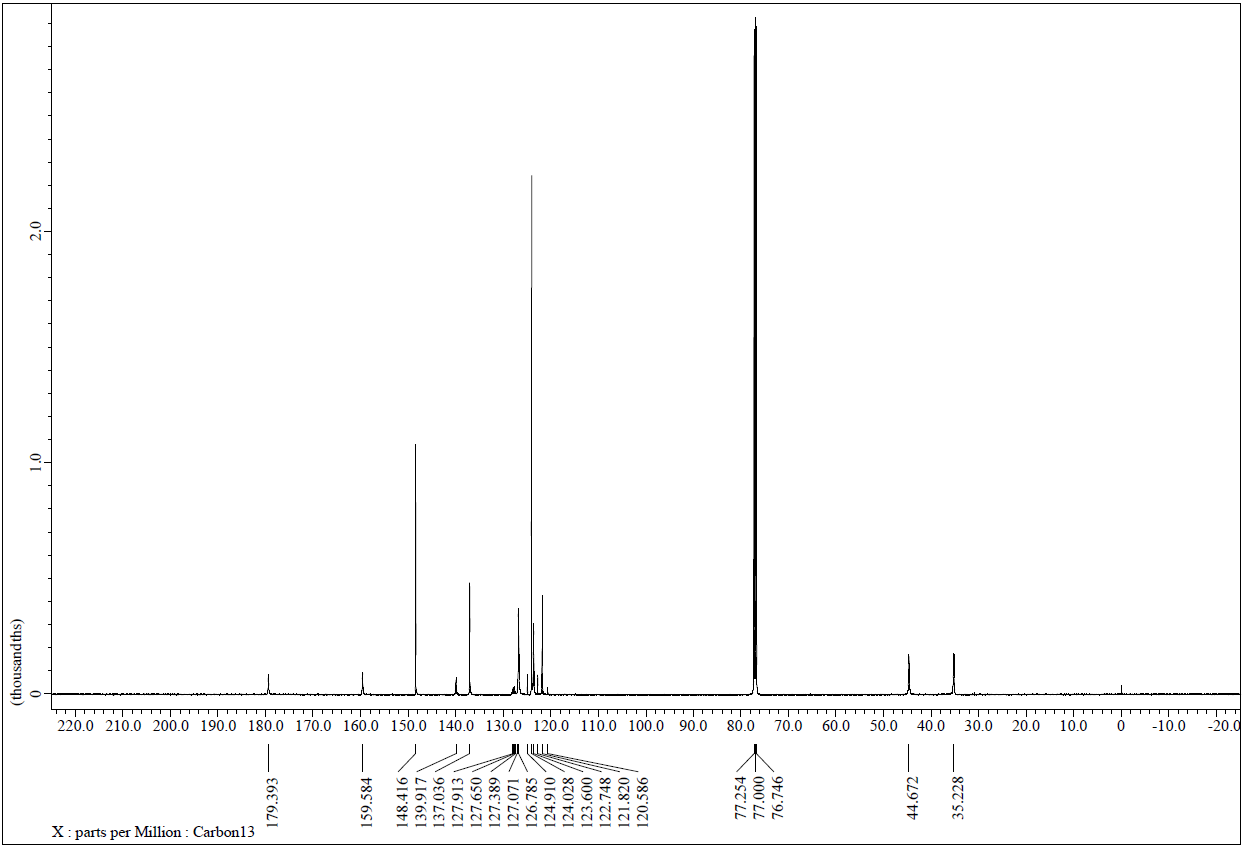


**Figure S6.** ^13^C NMR spectrum for compound ZKT4.


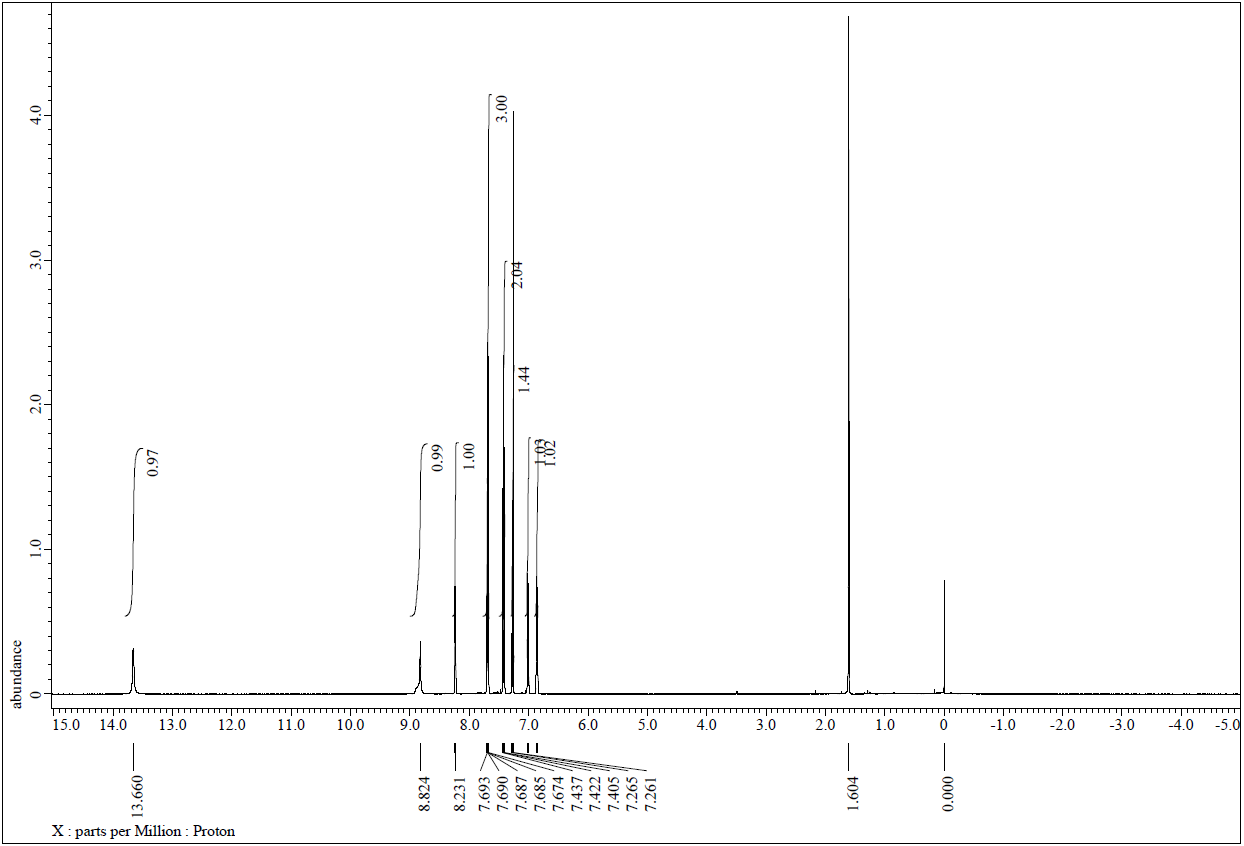


**Figure S7.** ^1^H NMR spectrum for compound ZKT5.


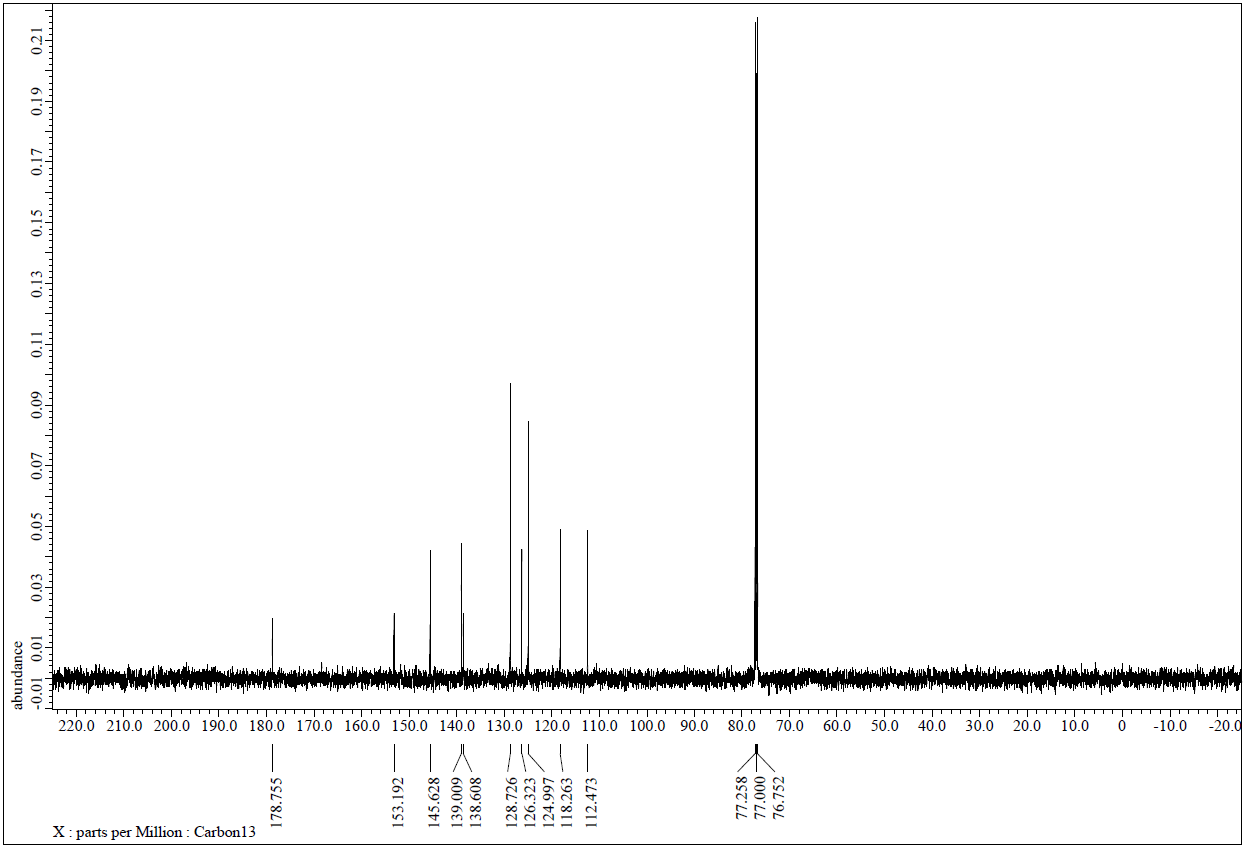


**Figure S8.** ^13^C NMR spectrum for compound ZKT5.


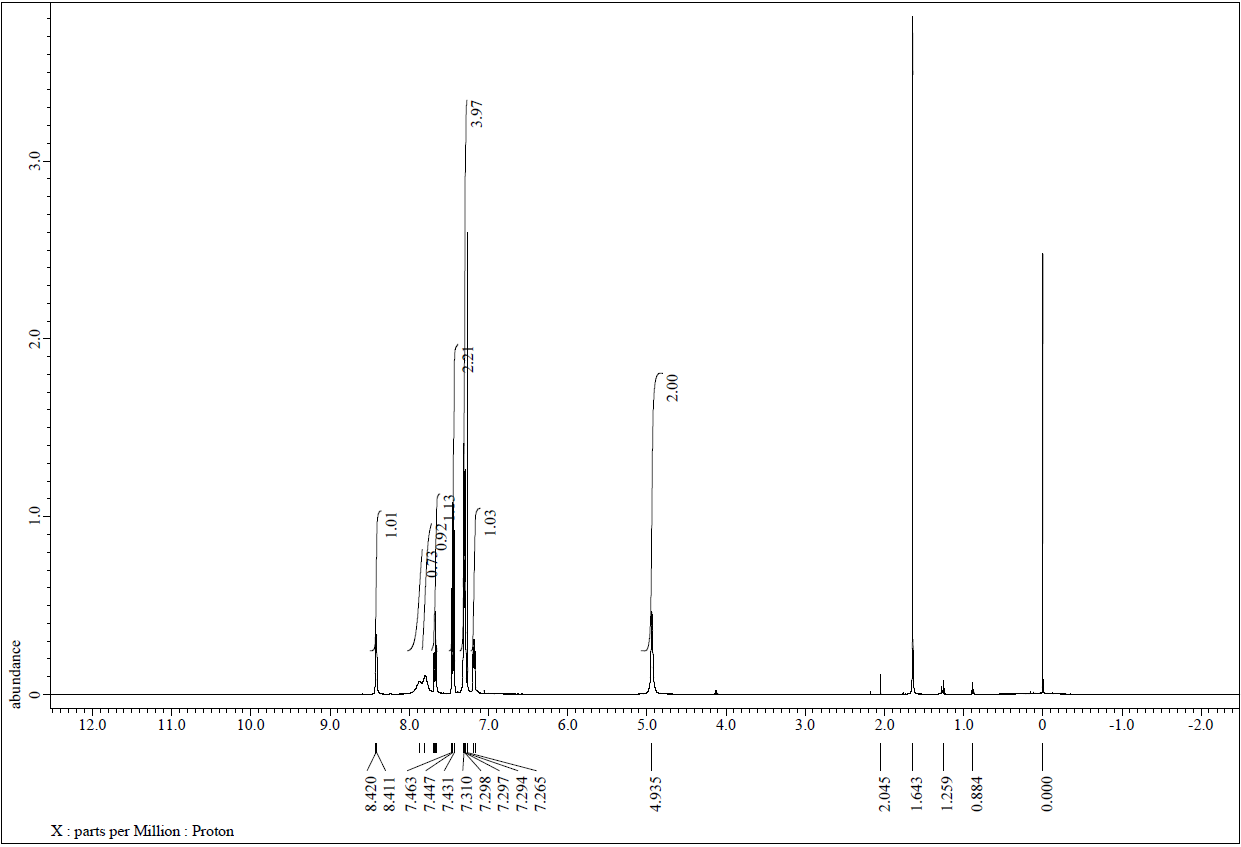


**Figure S9.** ^1^H NMR spectrum for compound ZKT6.


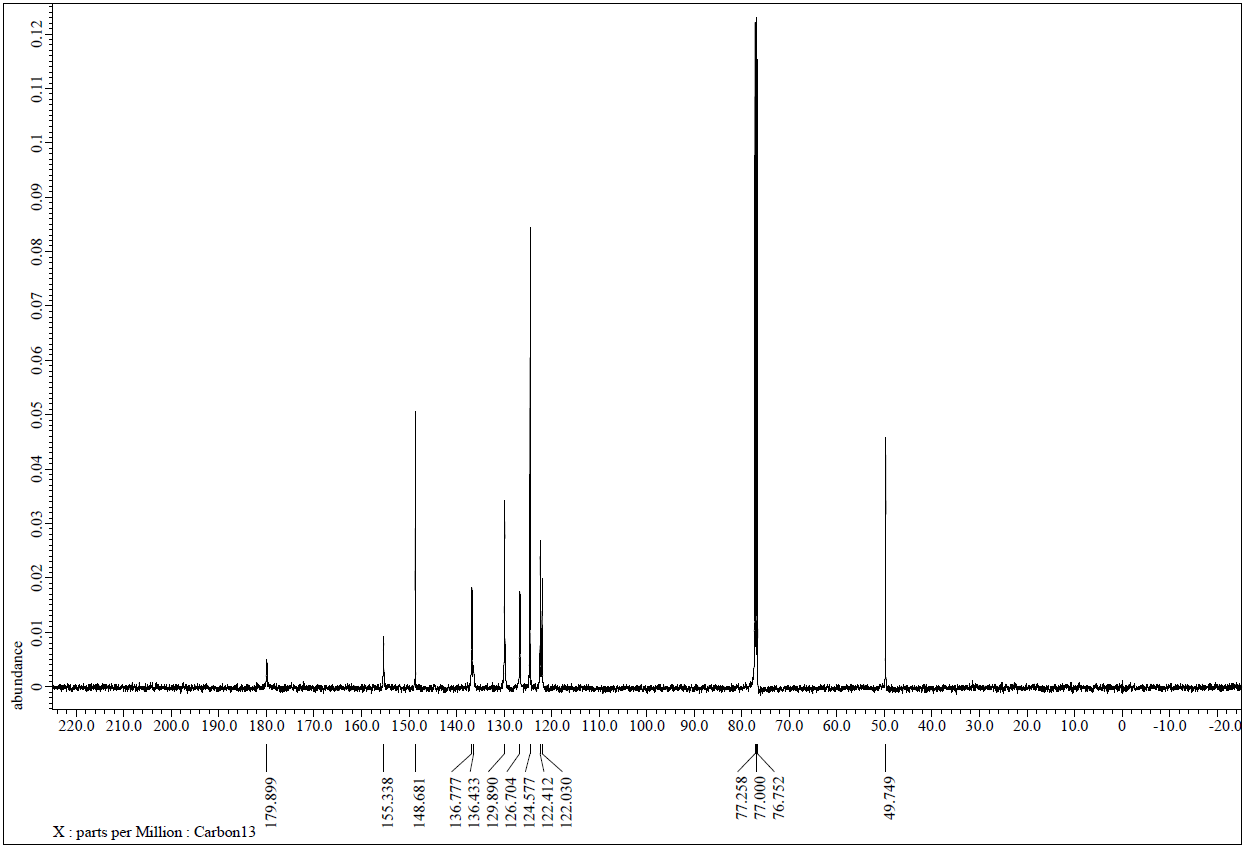


**Figure S10.** ^13^C NMR spectrum for compound ZKT6.


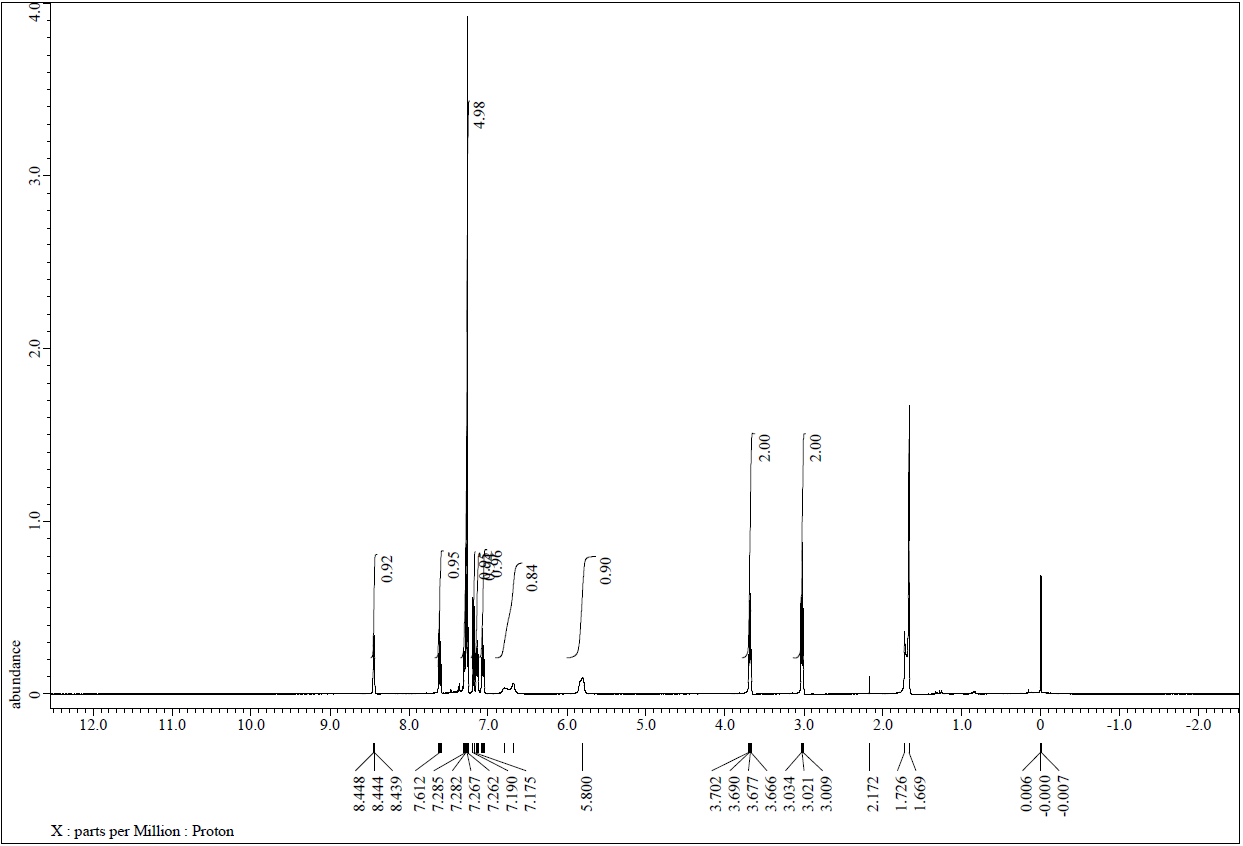


**Figure S11.** ^1^H NMR spectrum for compound ZKT7.


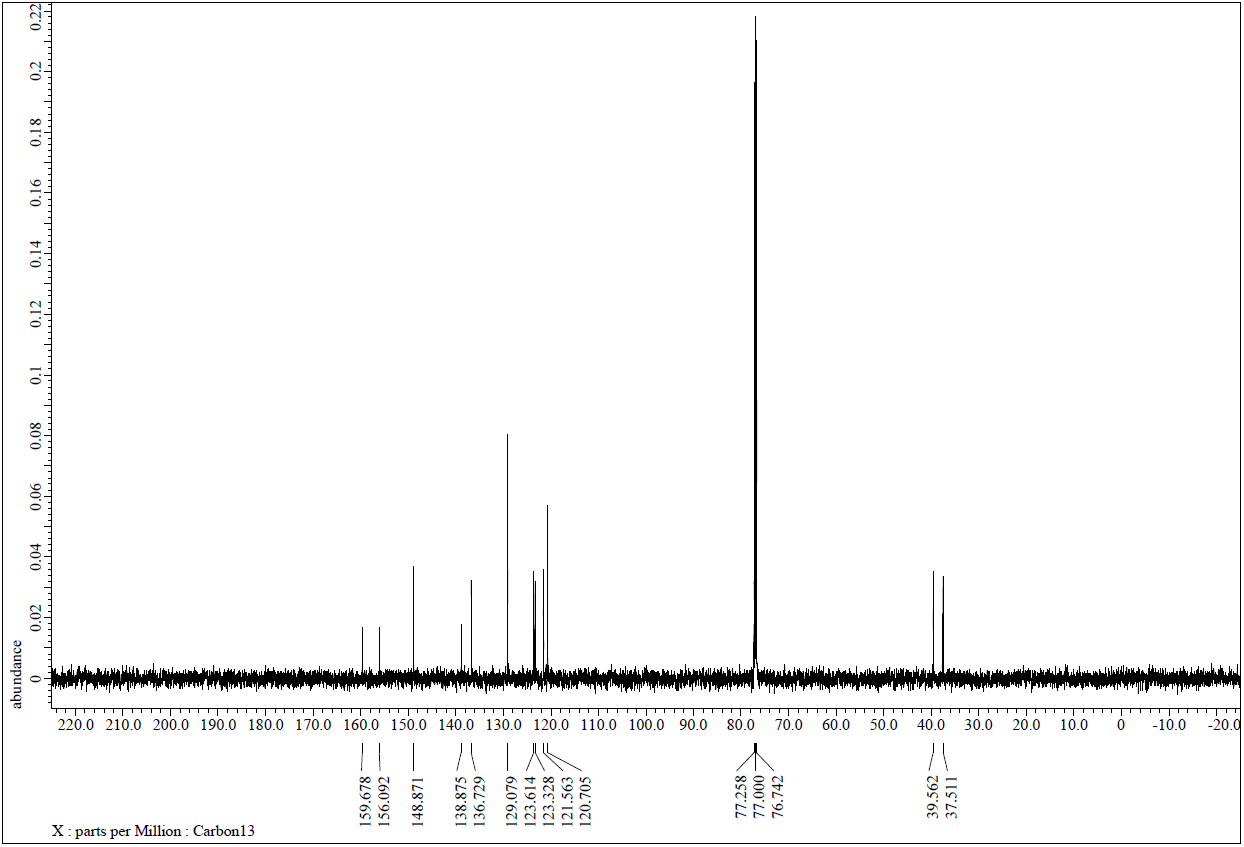


**Figure S12.** ^13^C NMR spectrum for compound ZKT7.


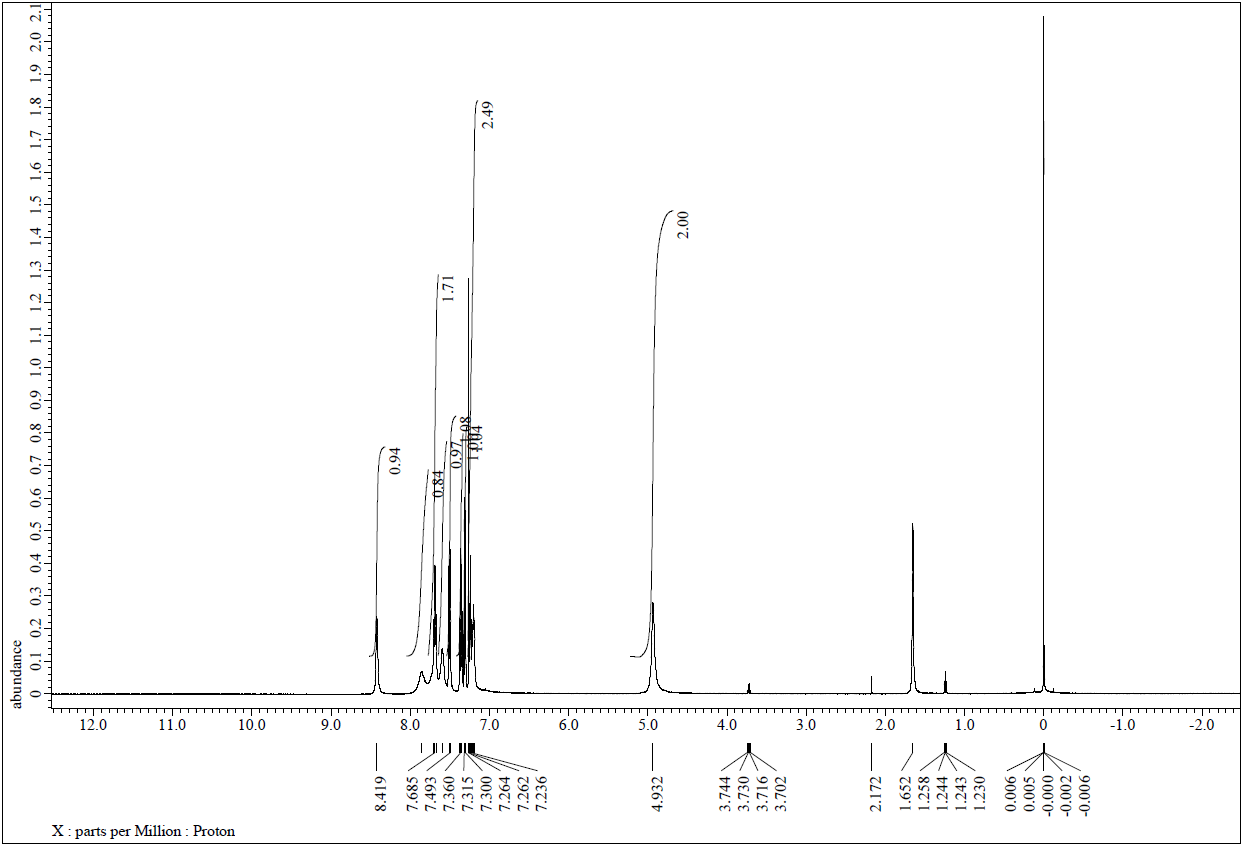


**Figure S13.** ^1^H NMR spectrum for compound ZKT8.


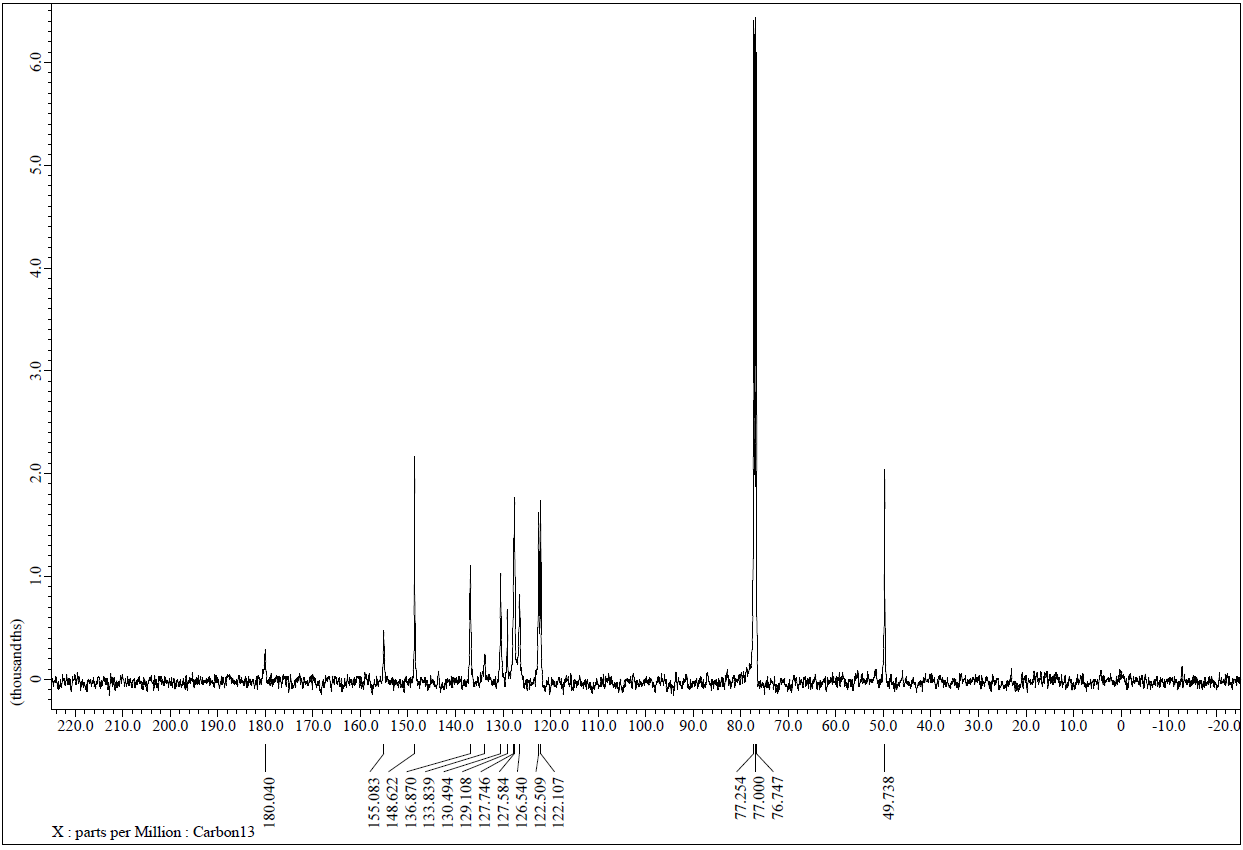


**Figure S14.** ^13^C NMR spectrum for compound ZKT8.


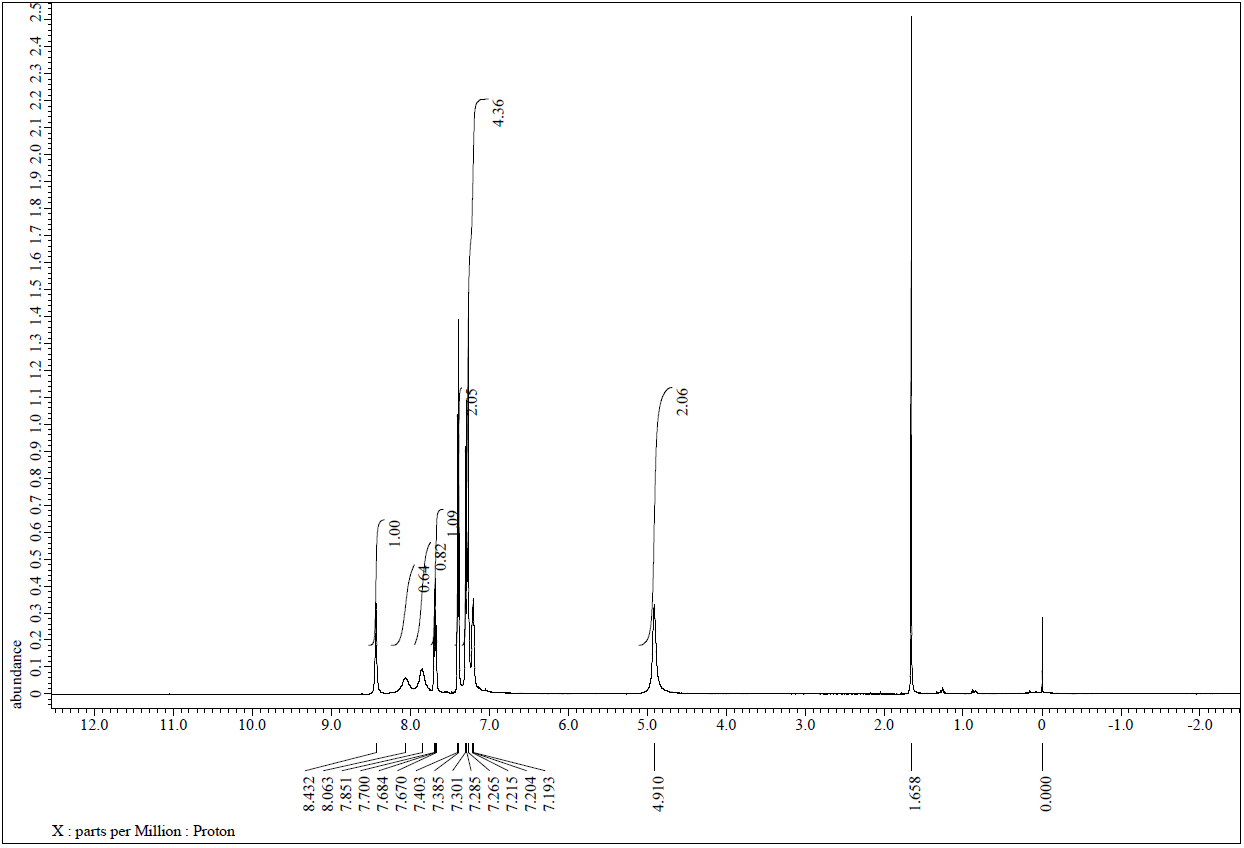


**Figure S15.** ^1^H NMR spectrum for compound ZKT9.


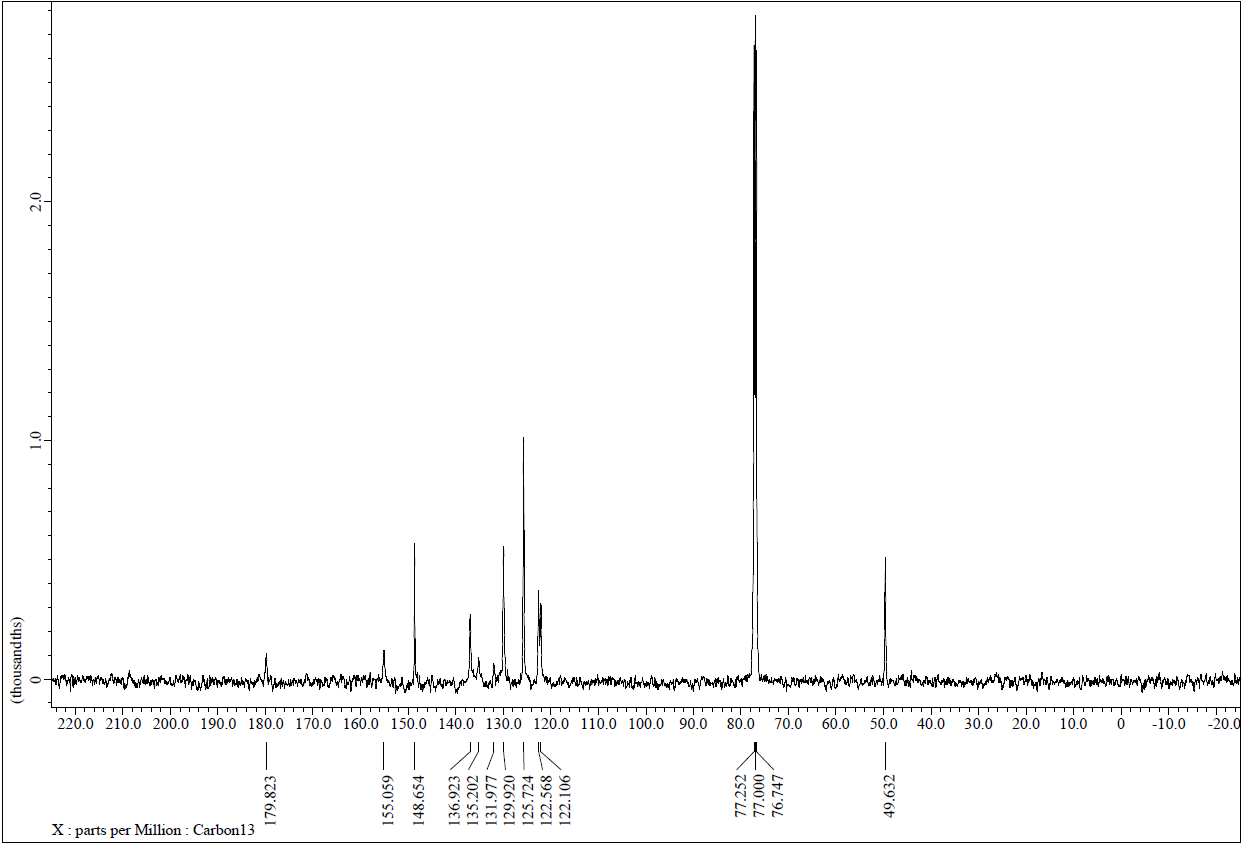


**Figure S16.** ^13^C NMR spectrum for compound ZKT9.


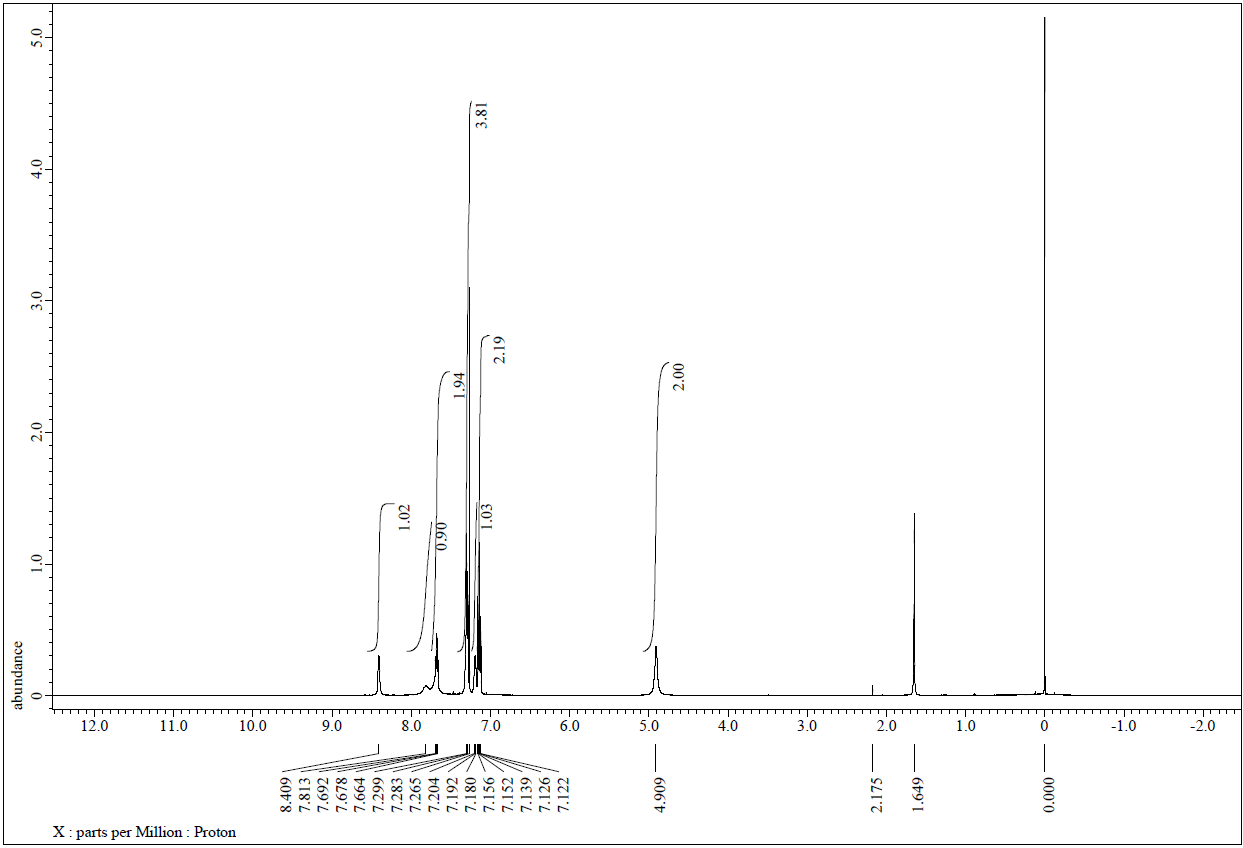


**Figure S17.** ^1^H NMR spectrum for compound ZKT10.


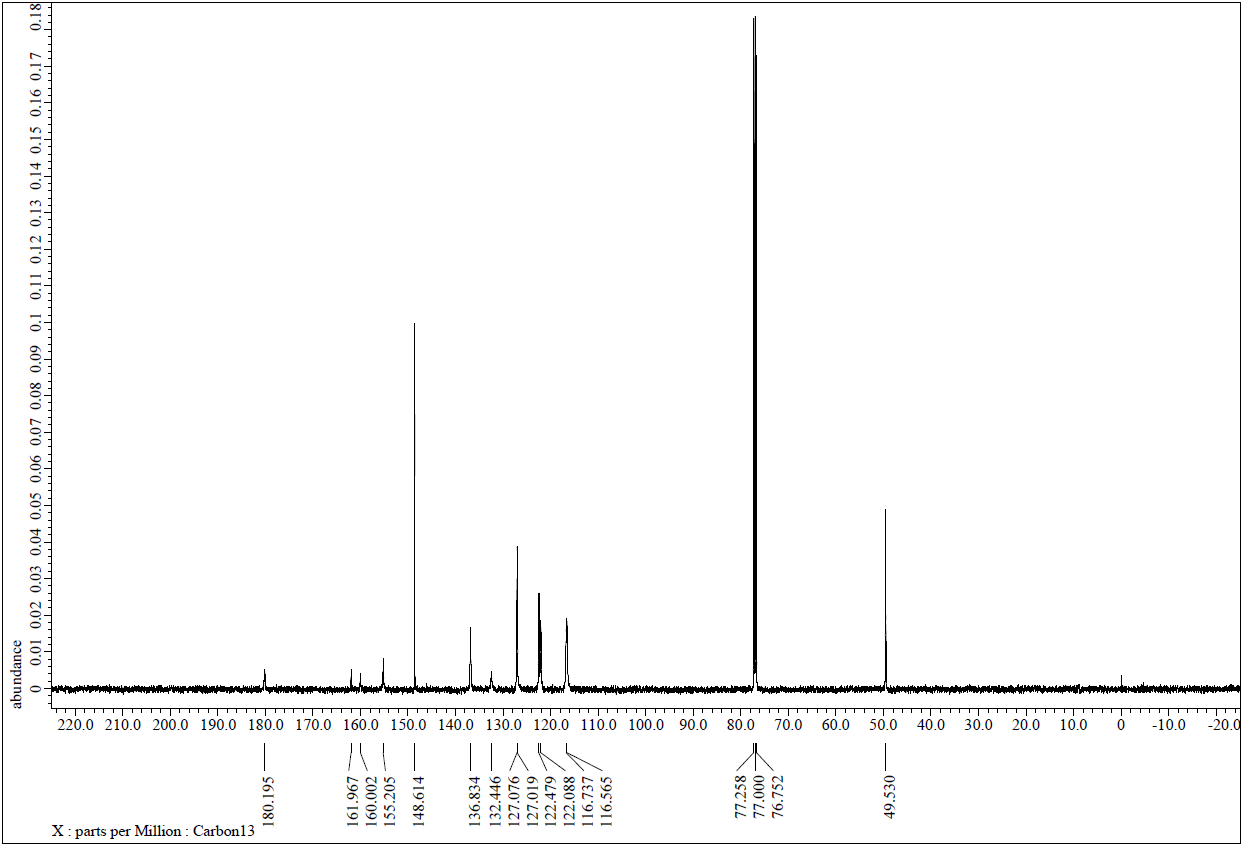


**Figure S18.** ^13^C NMR spectrum for compound ZKT10.


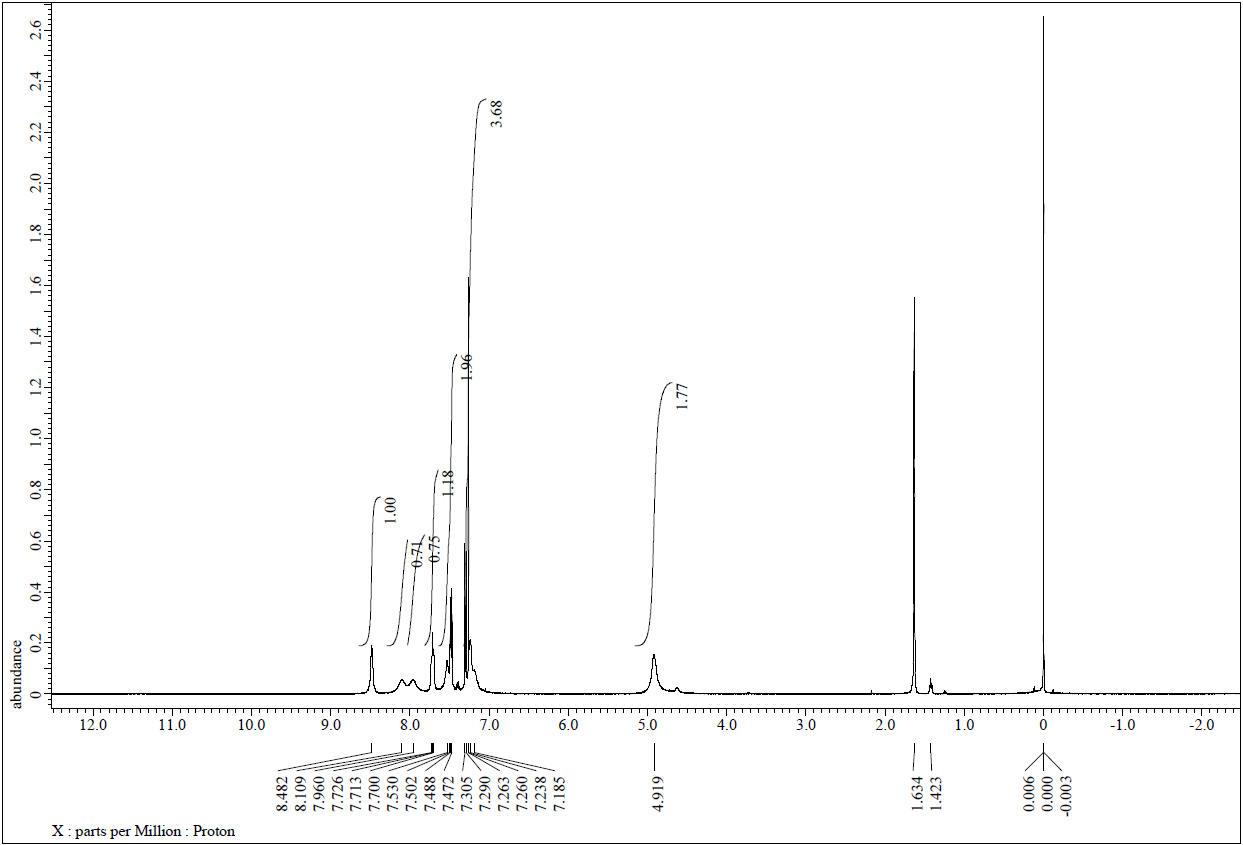


**Figure S19.** ^1^H NMR spectrum for compound ZKT11.


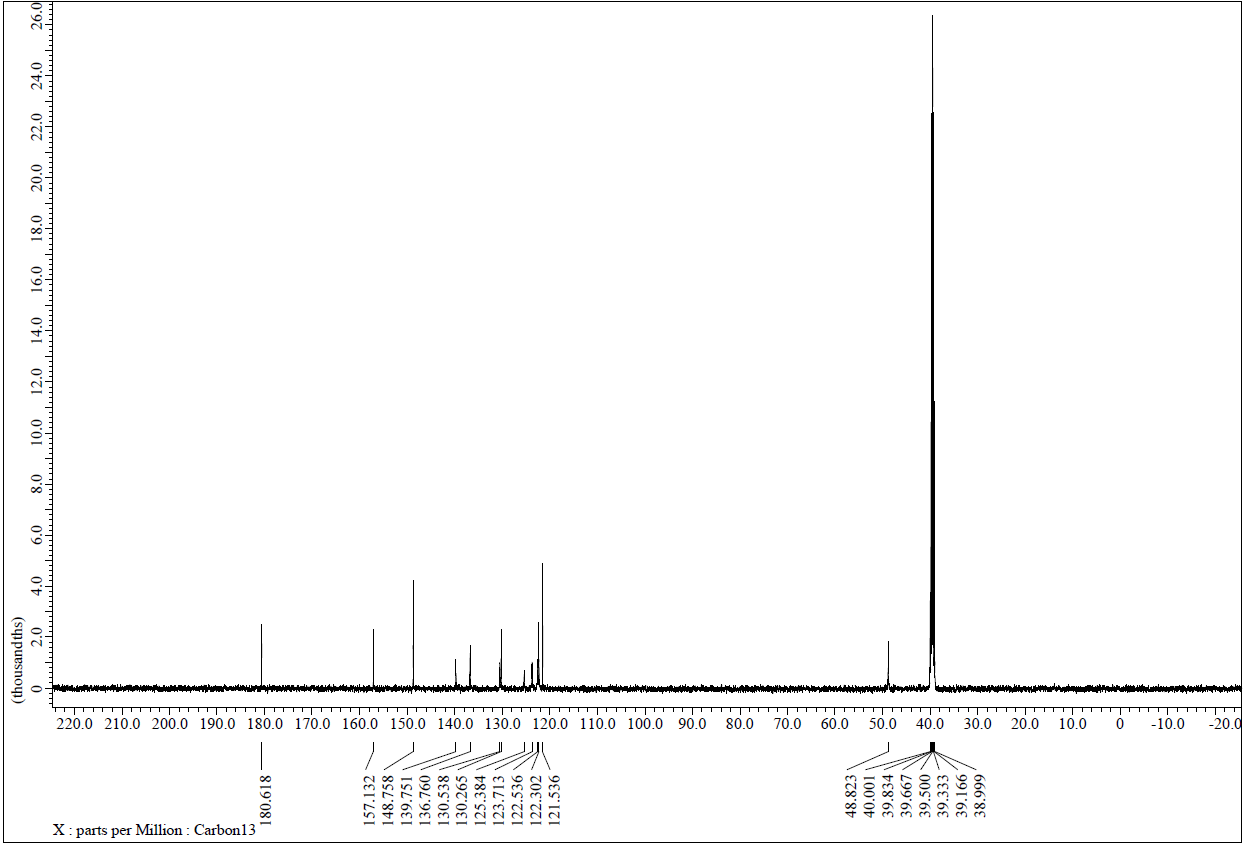


**Figure S20.** ^13^C NMR spectrum for compound ZKT11.


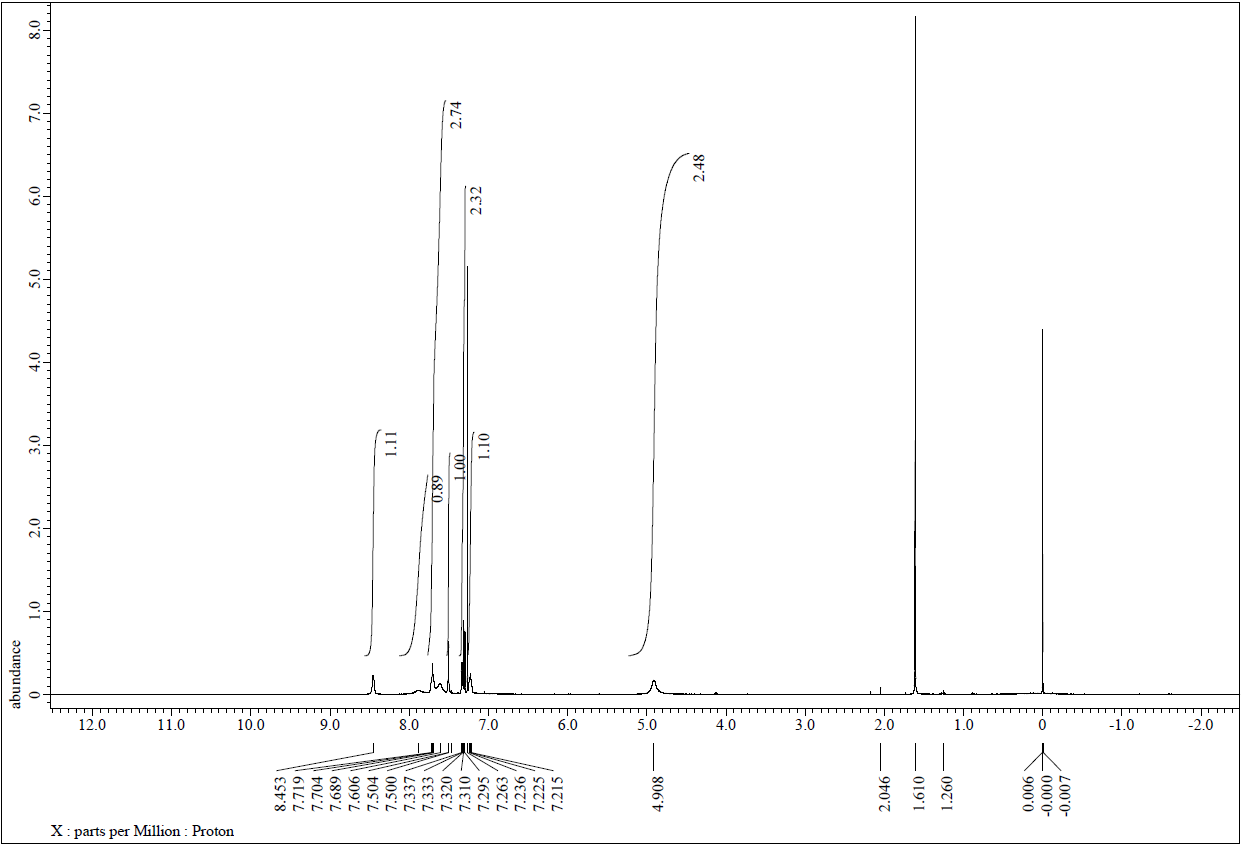


**Figure S21.** ^1^H NMR spectrum for compound ZKT12.


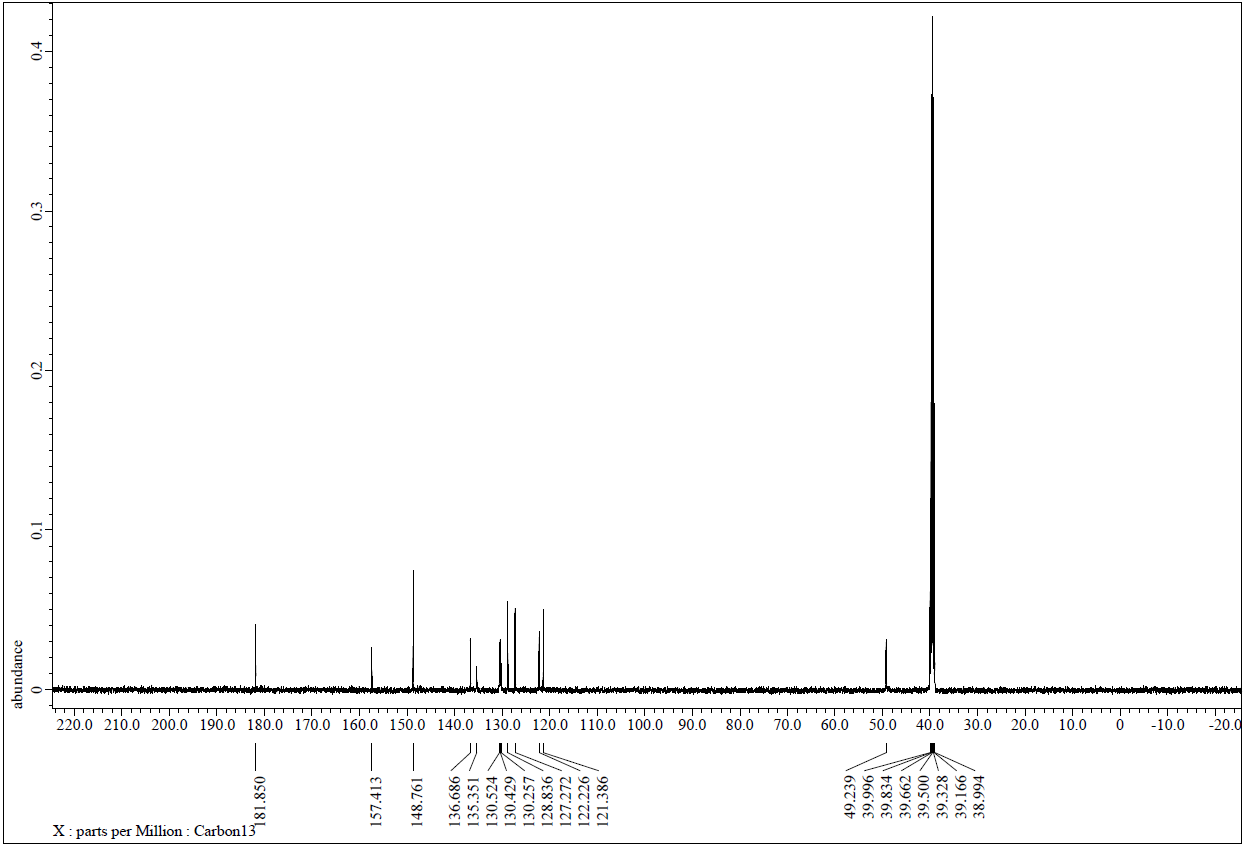


**Figure S22.** ^13^C NMR spectrum for compound ZKT12.


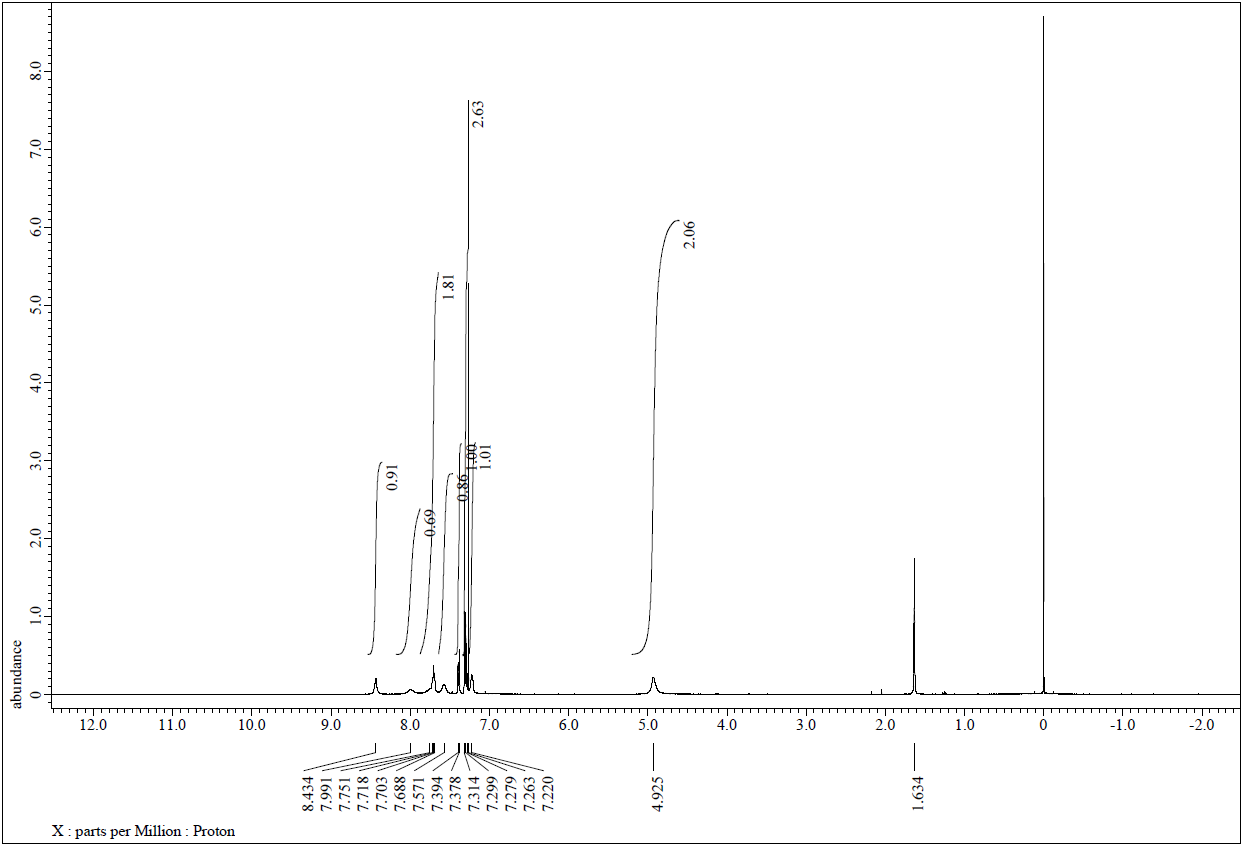


**Figure S23.** ^1^H NMR spectrum for compound ZKT13.


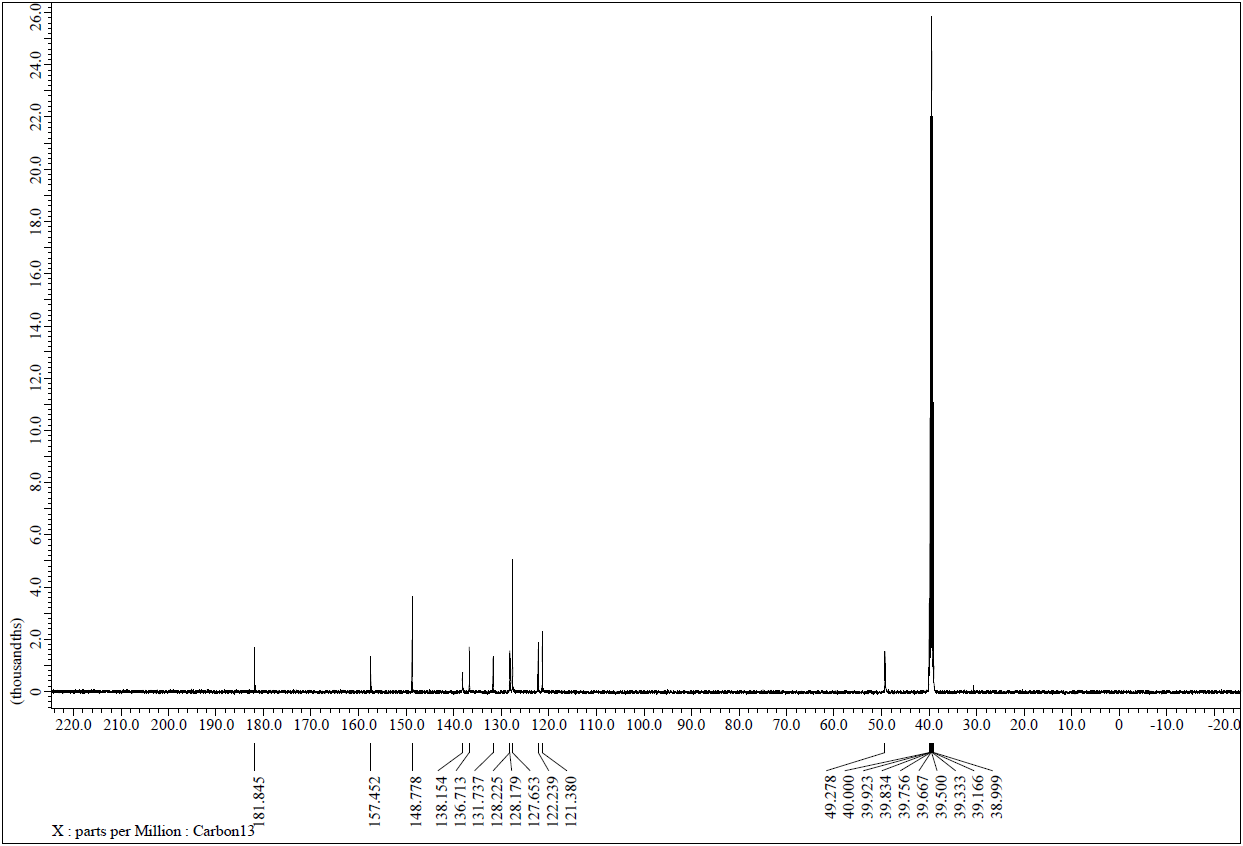


**Figure S24.** ^13^C NMR spectrum for compound ZKT13.


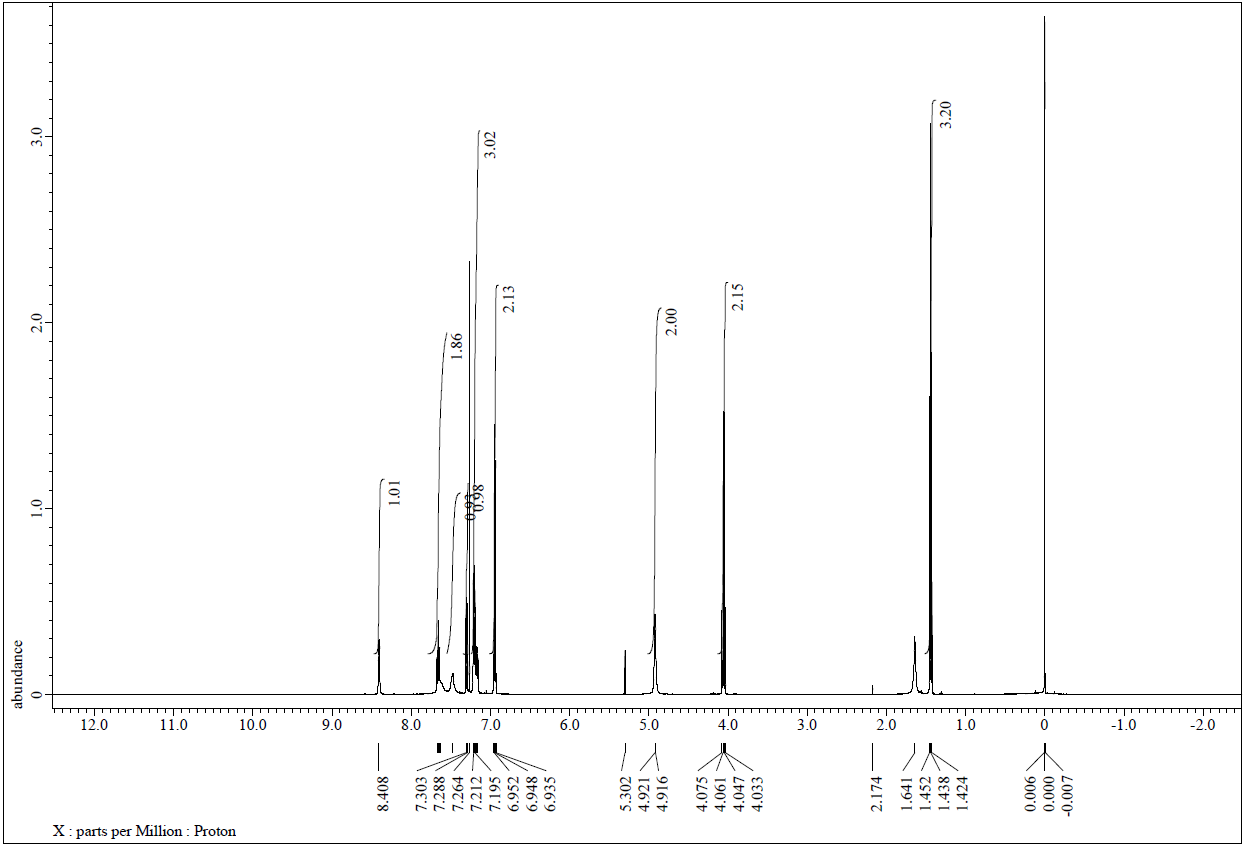


**Figure S25.** ^1^H NMR spectrum for compound ZKT14.


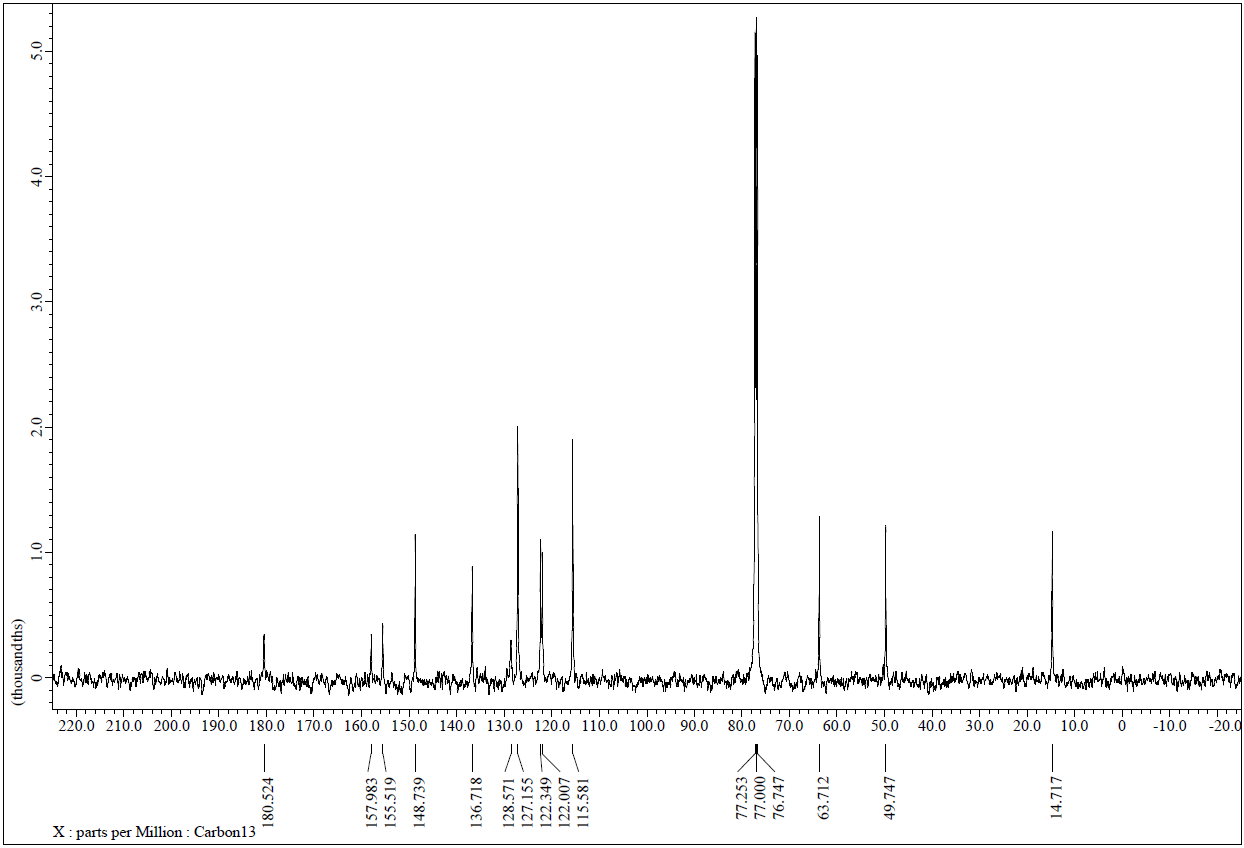


**Figure S26.** ^13^C NMR spectrum for compound ZKT14.


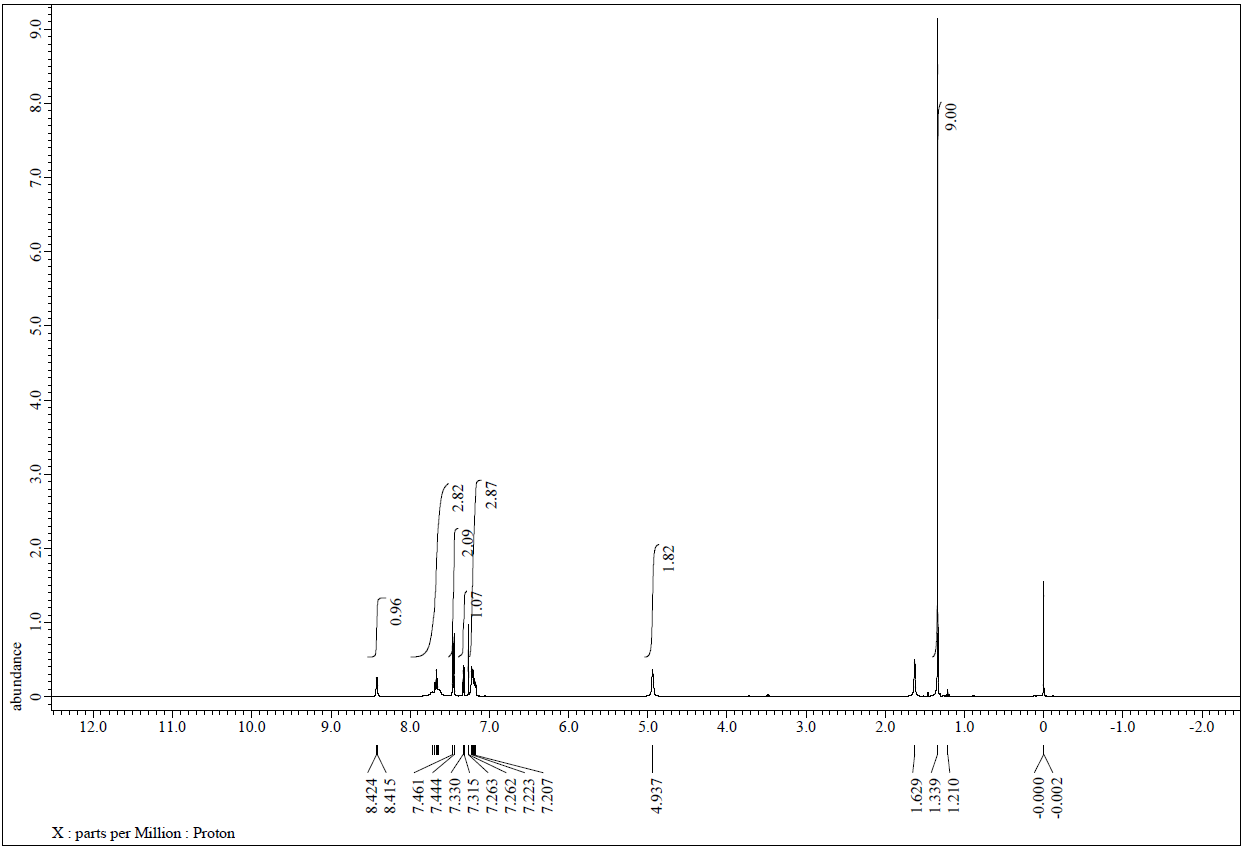


**Figure S27.** ^1^H NMR spectrum for compound ZKT15.


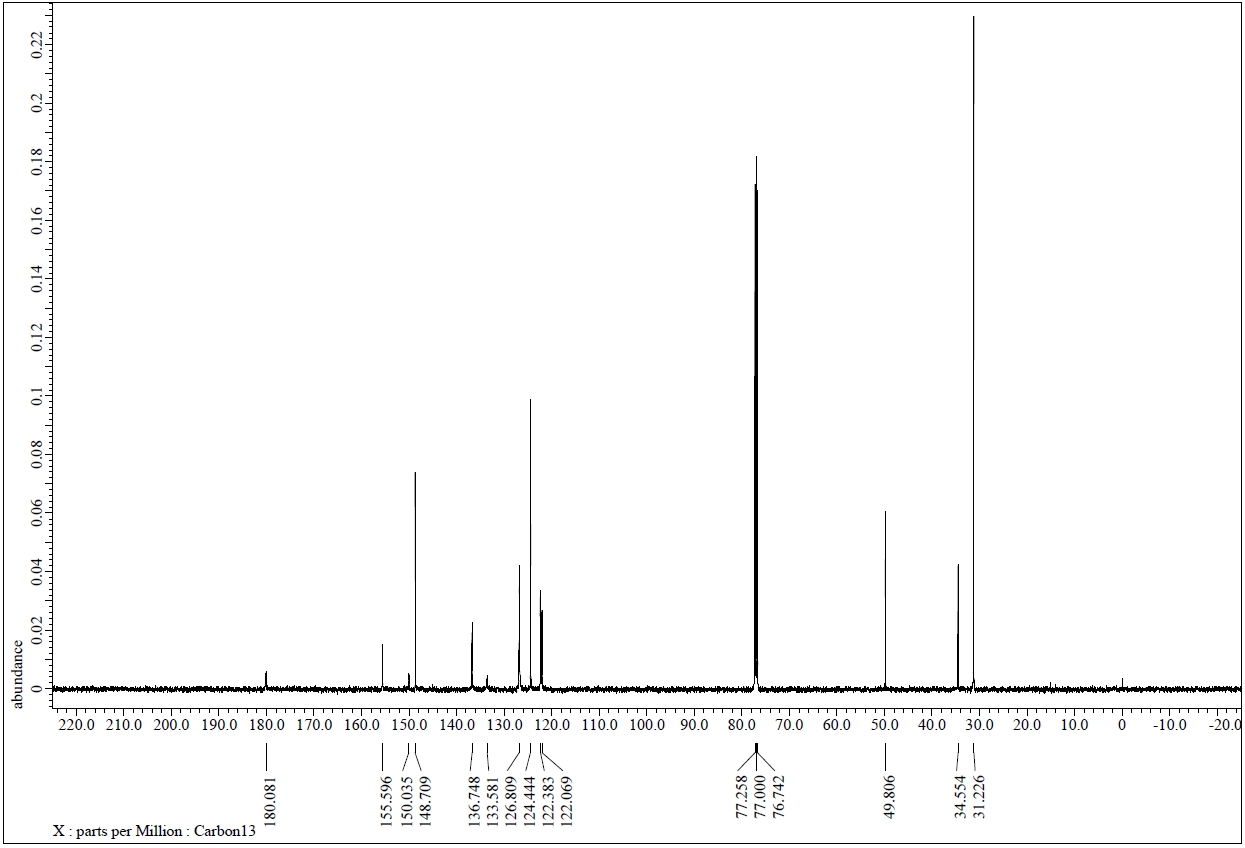


**Figure S28.** ^13^C NMR spectrum for compound ZKT15.


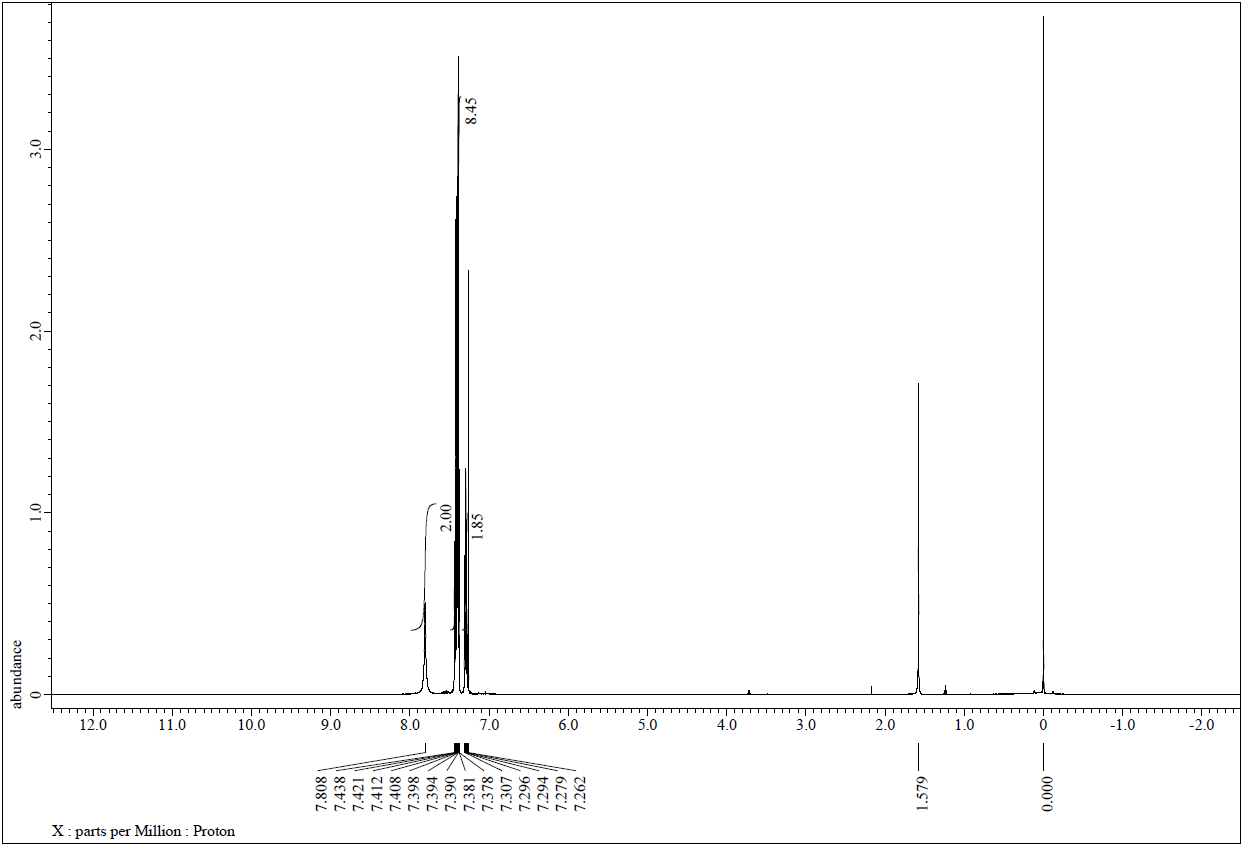


**Figure S29.** ^1^H NMR spectrum for compound ZKT16.


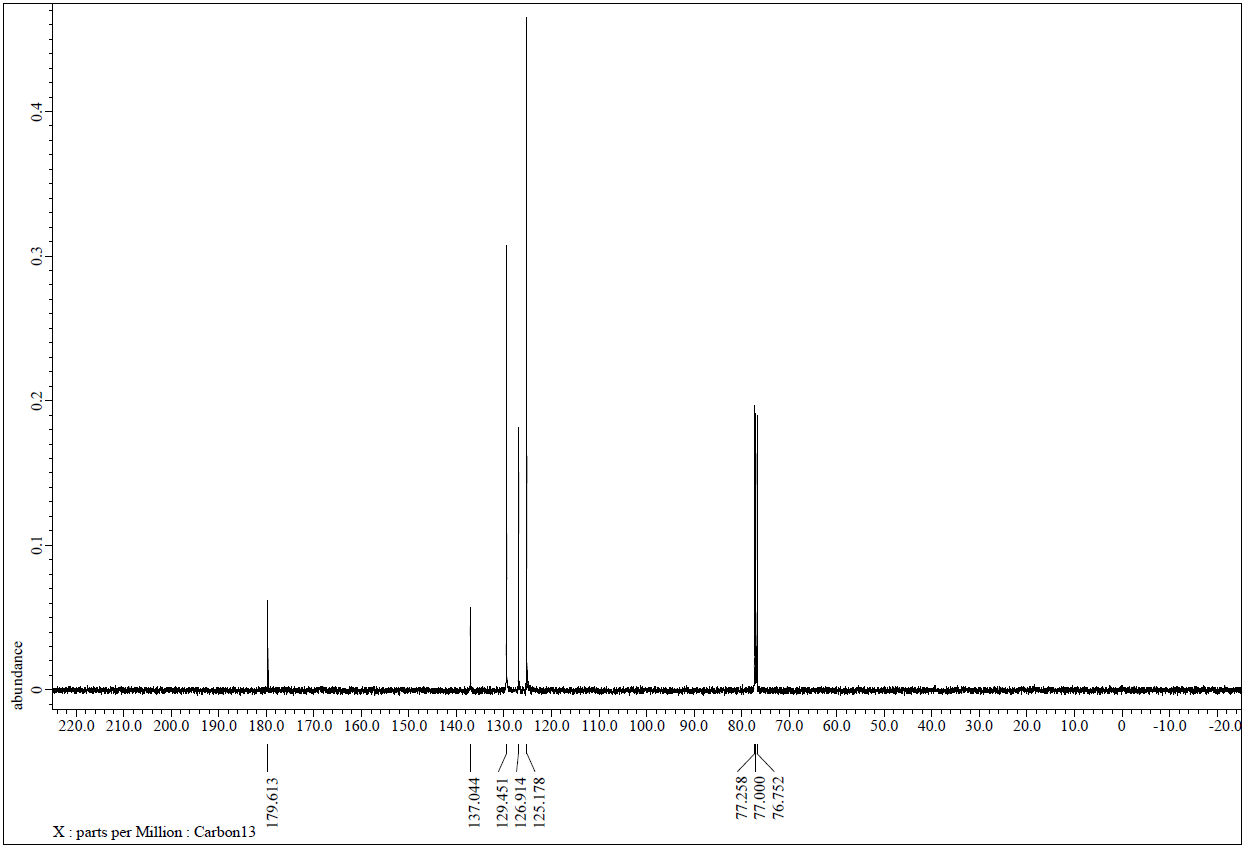


**Figure S30.** ^13^C NMR spectrum for compound ZKT16.


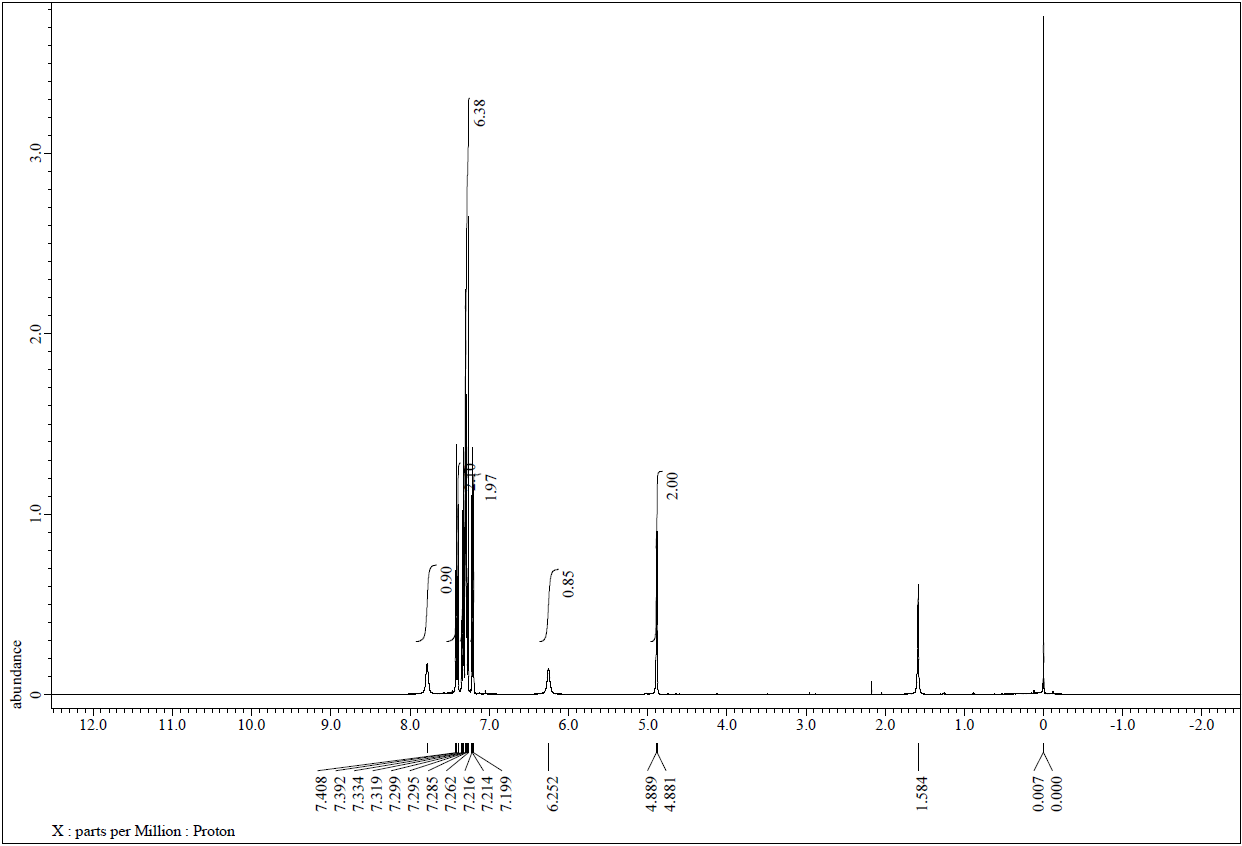


**Figure S31.** ^1^H NMR spectrum for compound ZKT17.


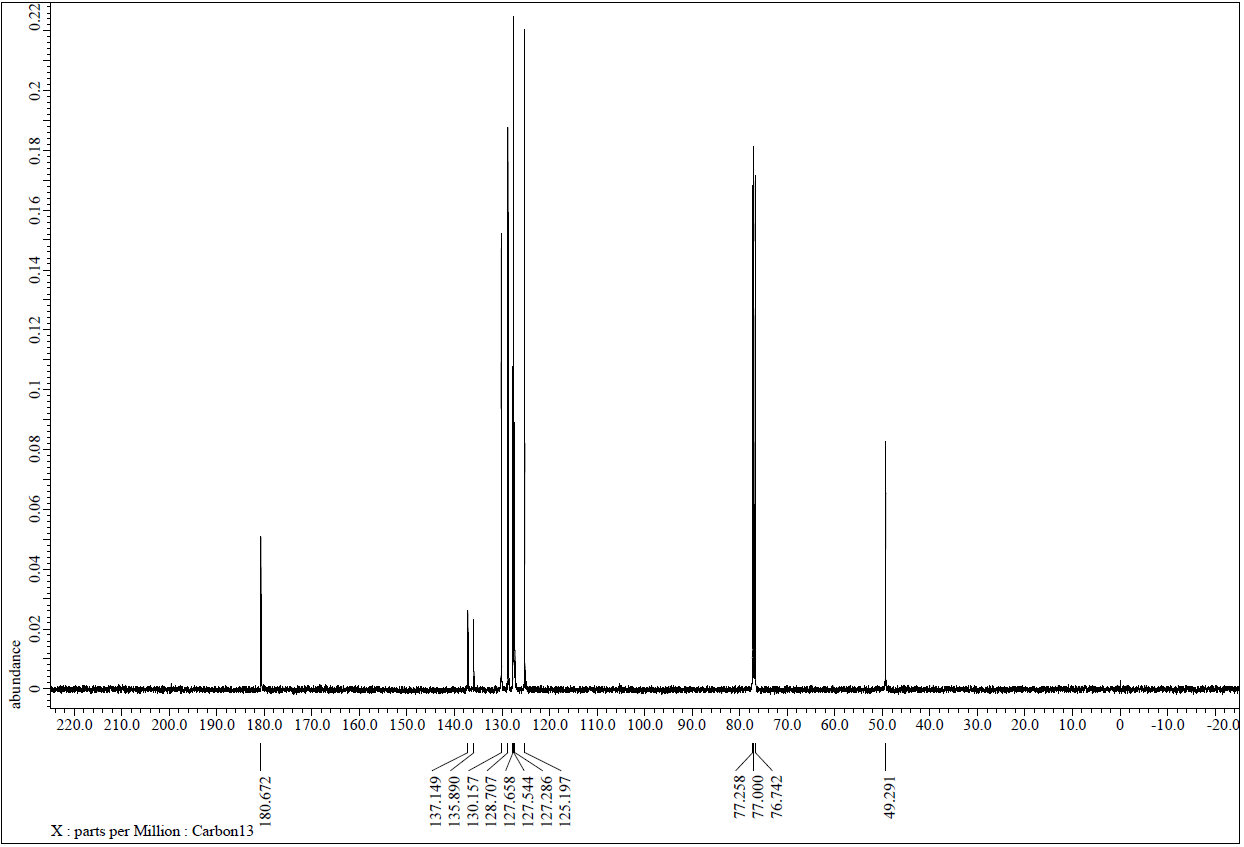


**Figure S32.** ^13^C NMR spectrum for compound ZKT17.


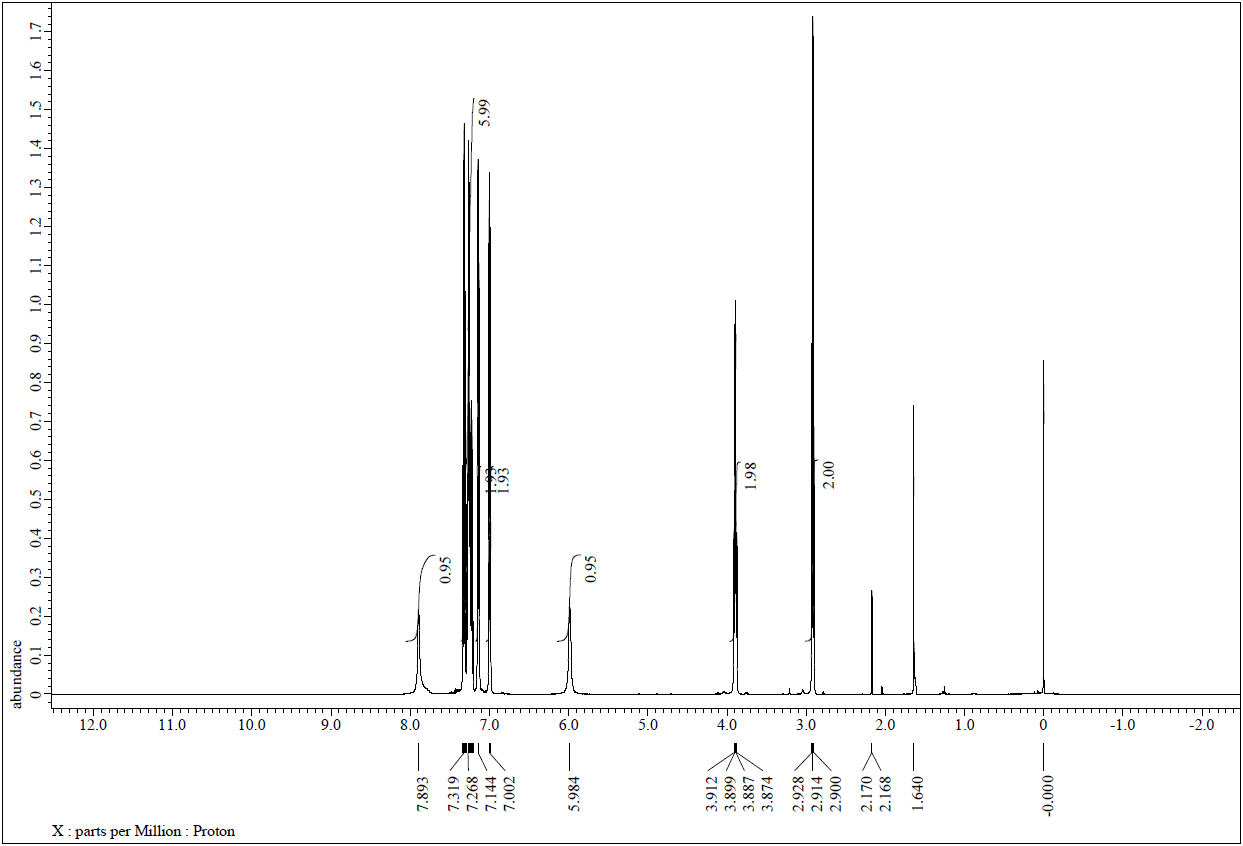


**Figure S33.** ^1^H NMR spectrum for compound ZKT18.


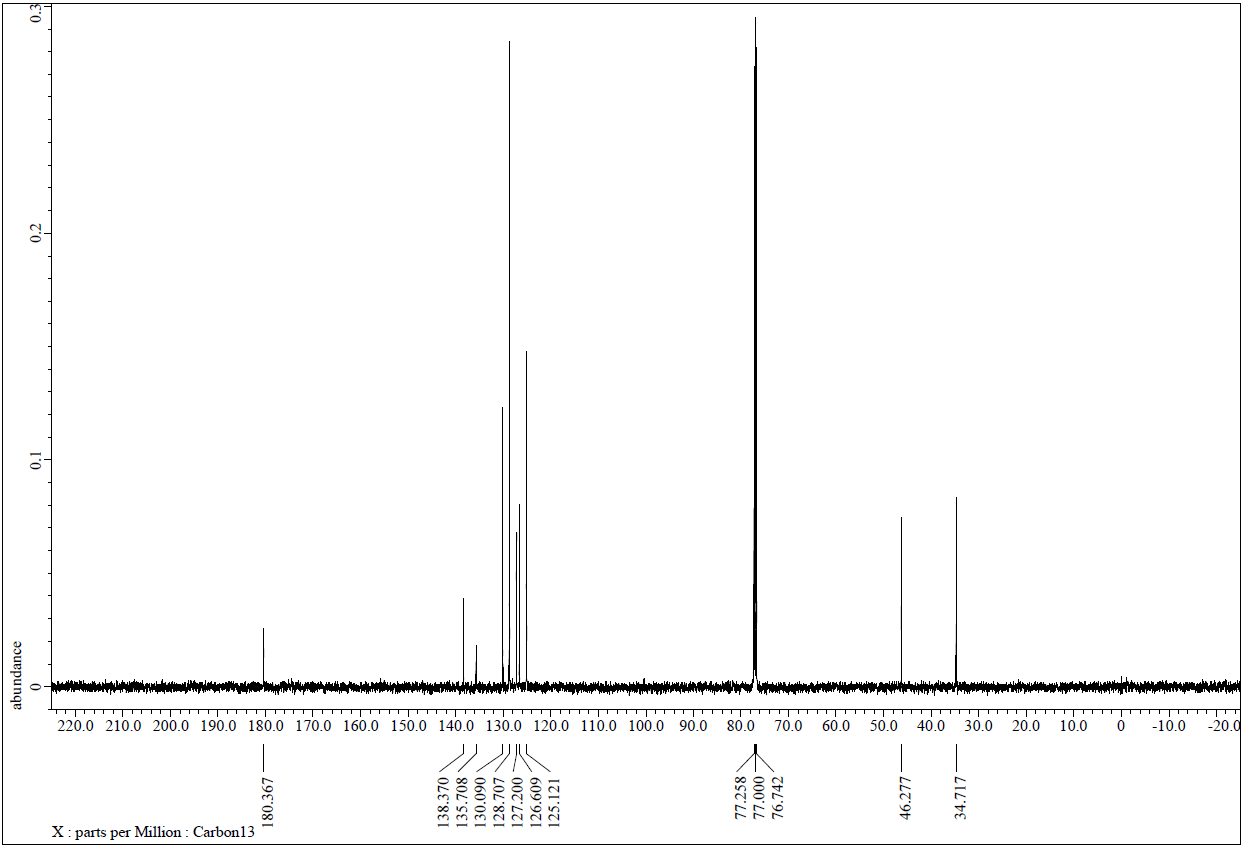


**Figure S34.** ^13^C NMR spectrum for compound ZKT18.
